# Pharmacological targets and mechanisms of calycosin against meningitis

**DOI:** 10.18632/aging.103886

**Published:** 2020-10-08

**Authors:** Yuan Nong, Yujia Liang, Xiaoliu Liang, Yongming Li, Bin Yang

**Affiliations:** 1 Department of Neurology (Area Two), Guigang City People’s Hospital, The Eighth Affiliated Hospital of Guangxi Medical University, Guigang, PR China; 2 College of Pharmacy, Guangxi Medical University, Nanning, Guangxi, PR China; 3 Department of Gynecology, Guigang City People's Hospital, The Eighth Affiliated Hospital of Guangxi Medical University, Guigang, Guangxi, PR China

**Keywords:** calycosin, meningitis, network pharmacology, biotargets and mechanisms

## Abstract

This report aimed to identity the potential anti-meningitis targets and mechanisms functioned by calycosin through network pharmacology approach. The bioinformatics databases were used to screen and collect the candidate genes/targets of calycosin and meningitis prior to identification of vital biotargets of calycosin-anti-meningitis. Additionally, the functional processes, signaling pathways of calycosin-anti-meningitis were screened and identified before further data visualization. As a result, all candidate and mapped biotargets of calycosin and meningitis were harvested before the vital targets of epidermal growth factor receptor (EGFR), tumor necrosis factor (TNF), epidermal growth factor (EGF), ataxia telangiectasia mutated protein (ATM), estrogen receptor alpha (ESR1), caspase-8 (CASP8), nerve growth factor (NGF) of calycosin-anti-meningitis were identified. The molecular processes of calycosin-anti-meningitis were screened and identified, including reduction of inflammatory development. Furthermore, the molecular pathways of calycosin-anti-meningitis were revealed, including suppression of NF-kappa B, Toll-like receptor, TNF signaling pathways. Molecular docking findings uncovered the docking capacity of calycosin with meningitis and potential pharmacological activity of calycosin against meningitis. In conclusion, these bioinformatic data uncovered the network targets and mechanisms of calycosin-anti-meningitis. And the current findings indicated that the vital targets might be used as potent biomarkers for detecting meningitis.

## INTRODUCTION

Meningitis is a serious disease featured with infection-induced diffusivity inflammation in the brain. Commonly, the meningitis may be caused by viral or bacterial infection, and the clinical symptoms may include headache, nausea and fever [1]. The onset of meningitis can be acute and chronic, and potential syndrome of acute stage will progress from hours to days [2]. In some countries including underdeveloped Africa, the morbidity of bacterial-induced meningitis is increasing yearly due to poor living condition [3]. In China, the mounting cases of acute meningitis has posed the economic burden for local government as the insufficient health-care occurs in outlying district [4]. The diagnosis of meningitis can have clinical manifestation and medical detection (blood, imaging tests), however, the bacterial meningitis therapy is clinically hard to cure due to the life-threatening complications [5]. In addition, the pathogenetic causes of meningitis are not entirely revealed so far. Accordingly, current anti-meningitis strategy warrants to be further explored through developing potential active ingredient. Historically, Chinese herbal medicine may be applied for effective treatment of endemic disease, such as gout [6]. Calycosin, rich in a Chinese herb of *Radix astragali*, is experimentally evidenced with some pharmacological activities, such as anti-cancers, anti-aging [7, 8]. Furthermore, neuroprotective action of calycosin is reported in preclinical study [9]. However, anti-meningitis effect exerted by calycosin has not been reported. An applicable tool of network pharmacology is recently established for screening candidate genes/targets, and uncovering functions and curative mechanisms of potential active ingredient against disease [10, 11]. Interestingly, our previous reports have revealed the biotarget and therapeutic mechanisms of vitamin C against liver injury, and plumbagin to treat colorectal cancer [12, 13]. In this bioinformatics study, we used a promising approach of network pharmacology to screen out the candidate biotarget, and characterize the functions and pharmacological mechanisms of calycosin-anti-meningitis before the bioinformatics findings would be validated in our future experiments.

## RESULTS

### Predictive genes/targets of calycosin and meningitis

**Figure 1:**
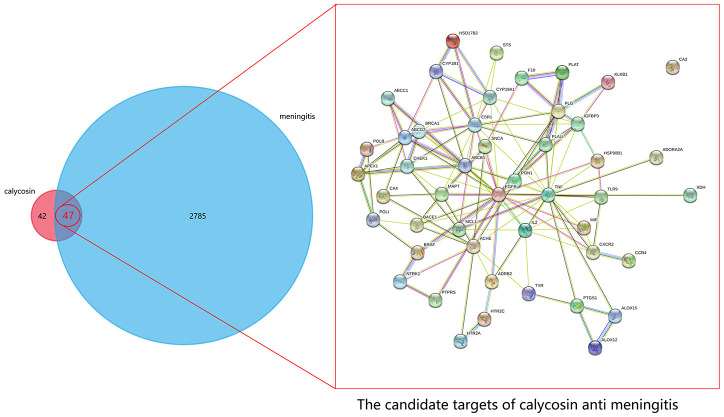
**All genes of calycosin and meningitis were screened and identified prior to further identification of the mapping targets of calycosin-anti-meningitis.**

Following the bioinformatics assays, a number of 2832 meningitis-associated genes/targets were screened and obtained. The 89 well-reported genes/targets of calycosin were collected by using the online-based databases. And another 47 mapped targets of calycosin-anti-meningitis were identified by use of the FunRich software. Subsequently, other 186 targets were harvested from the STRING database based on the minimum required interaction score (0.04), and a PPI network of calycosin-anti-meningitis were plotted, as showed in Figure 1.

### Topology parameters in PPI network from vital targets

**Figure 2:**
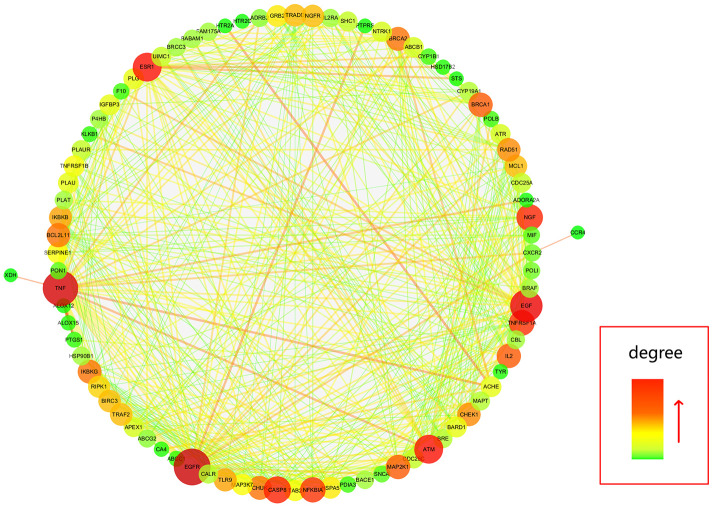
**All candidate targets of calycosin-anti-meningitis were collected for construction of a PPI network.**

**Figure 3:**
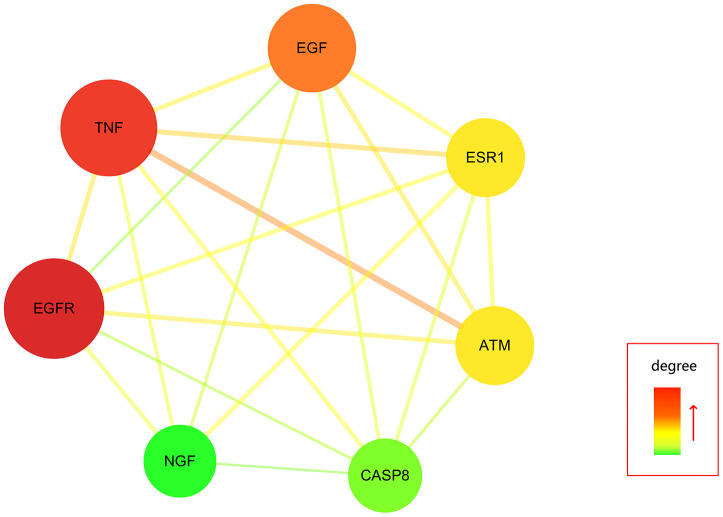
**All vital targets of calycosin-anti-meningitis was screened and identified.**

Further, all mapped intersection genes were imported to Cytoscape, and then the topological parameters of calycosin-anti-meningitis were determined and reported by using NetworkAnalyzer assays, as shown in candidate genes for data visualization (Figure 2). Following with the assays of Network Statistics and Simple Parameters, the average degree of freedom calculated in the target was 12.837, and the maximum degree of freedom was 49. Therefore, the range of vital target screening conditions was designed as from 26 to 49, and then 7 vital targets were obtained, showing as EGFR, TNF, EGF, ATM, ESR1, CASP8, and NGF (Figure 3).

### Findings of biological functions and pathway enrichment of vital targets

**Figure 4:**
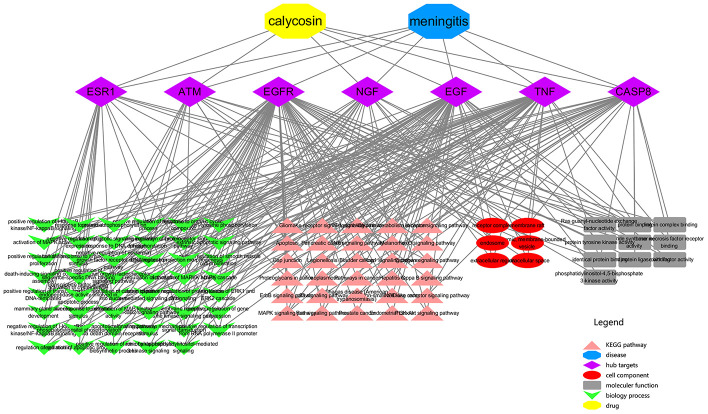
**An integrated network from vital targets was plotted and revealed the intersection association of target-disease-function-pathway in calycosin-anti-meningitis.**

**Figure 5:**
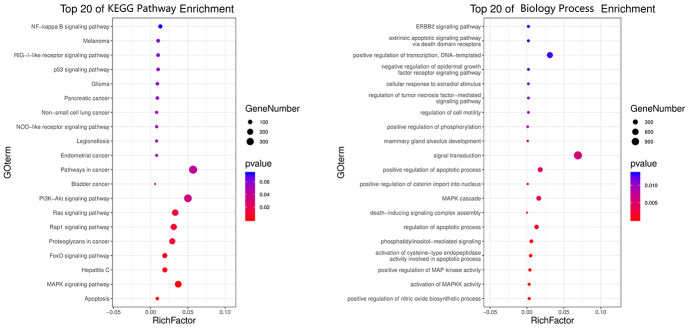
**All top 20 biological processes and molecular pathways of calycosin-anti- meningitis from enrichment analyses were revealed and visualized.**

**Figure 6:**
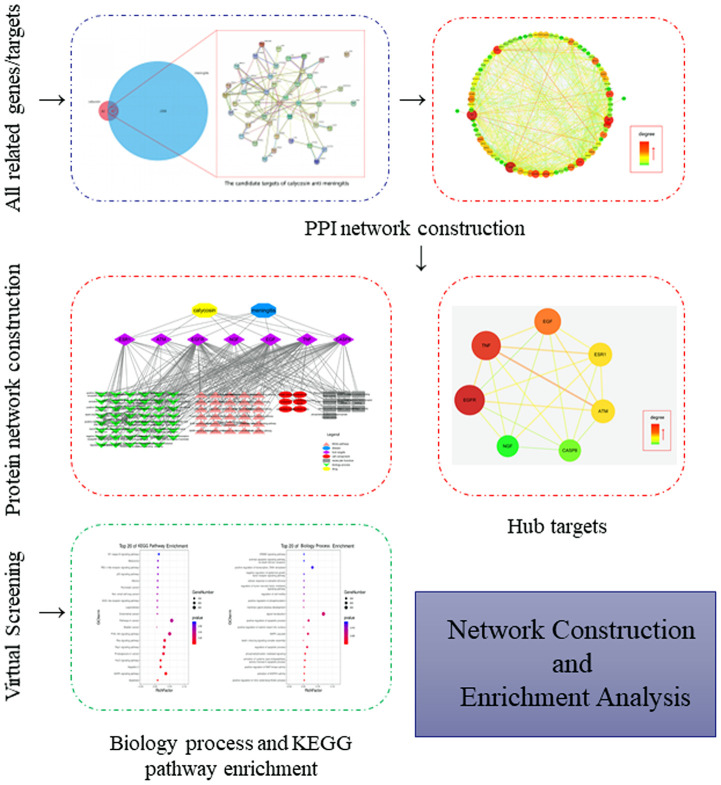
**A flow-chart using a network pharmacology approach aimed to reveal and characterize the biological targets, functions and molecular pathways in calycosin-anti-meningitis.**

All top KEGG signaling pathway of the vital target were revealed and presented through the DAVID database, and then the important target-related pathway interaction network was showed in Figure 4. The primary signaling pathway of vital targets in calycosin-anti-meningitis were involved in effective regulation of Apoptosis, MAPK signaling pathway, Hepatitis C, FoxO signaling pathway, Proteoglycans in cancer, Rap1 signaling pathway, Ras signaling pathway, PI3K-Akt signaling pathway, Bladder cancer, Pathways in cancer, Endometrial cancer, Legionellosis, NOD-like receptor signaling pathway, Non-small cell lung cancer, Pancreatic cancer, Glioma, p53 signaling pathway, RIG-I-like receptor signaling pathway, Melanoma, NF-kappa B signaling pathway, ErbB signaling pathway, Prostate cancer, Gap junction, HIF-1 signaling pathway, Estrogen signaling pathway, Choline metabolism in cancer, Chagas disease (American trypanosomiasis), Toll-like receptor signaling pathway, TNF signaling pathway, Toxoplasmosis (more details shown in Supplementary Figure 1). Furthermore, the biological functions of vital targets were identified through the DAVID database, as revealed in positive regulation of nitric oxide biosynthetic process, activation of MAPKK activity, positive regulation of MAP kinase activity, activation of cysteine-type endopeptidase activity involved in apoptotic process, phosphatidylinositol-mediated signaling, regulation of apoptotic process, death-inducing signaling complex assembly, MAPK cascade, positive regulation of catenin import into nucleus, positive regulation of apoptotic process, signal transduction, mammary gland alveolus development, positive regulation of phosphorylation, regulation of cell motility, regulation of tumor necrosis factor-mediated signaling pathway, cellular response to estradiol stimulus, negative regulation of epidermal growth factor receptor signaling pathway, positive regulation of transcription, DNA-templated, extrinsic apoptotic signaling pathway via death domain receptors, ERBB2 signaling pathway, negative regulation of I-kappaB kinase/NF-kappaB signaling, extrinsic apoptotic signaling pathway, cellular response to amino acid stimulus, intrinsic apoptotic signaling pathway in response to DNA damage, neuron projection morphogenesis, positive regulation of fibroblast proliferation, epidermal growth factor receptor signaling pathway, cellular response to organic cyclic compound, positive regulation of smooth muscle cell proliferation, cellular response to mechanical stimulus, regulation of phosphatidylinositol 3-kinase signaling, positive regulation of protein kinase B signaling, response to estradiol, phosphatidylinositol phosphorylation, transmembrane receptor protein tyrosine kinase signaling pathway, positive regulation of sequence-specific DNA binding transcription factor activity, activation of MAPK activity, positive regulation of transcription from RNA polymerase II promoter, positive regulation of protein phosphorylation, negative regulation of gene expression, peptidyl-tyrosine phosphorylation, DNA replication, positive regulation of I-kappaB kinase/NF-kappaB signaling, protein autophosphorylation, positive regulation of ERK1 and ERK2 cascade, positive regulation of gene expression, cell surface receptor signaling pathway (more details shown Supplementary Table 1). More markedly, the top 20 functional processes and molecular pathways of calycosin-anti-meningitis were optimized and highlighted (Figure 5). Collectively, we concluded that the bioinformatics flow-scheme by using network pharmacology was demonstrated in Figure 6.

### Molecular docking findings

**Figure 7:**
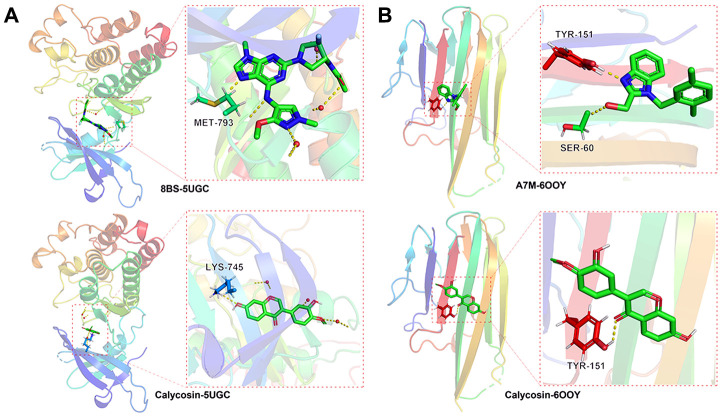
**Molecular docking data indicated that the binding capacity of calycosin with meningitis was significant in the vital targets of** EGFR, 8BS-5UGC (**A**), TNF, A7M-6OOY (**B**).

In the EGFR protein, the RMSD of the original ligand was 3.889 Å. The hydrogen bonding between the pro-ligand 8BS and the 5UGC protein acted on the amino acid residue with MET-793 (1.9Å), and the free binding energy with protein was -8.06 kcal/mol. Calycosin formed hydrogen bond with amino acid residue LYS-745 (2.4Å), and the free binding energy with protein is -8.9 kcal/mol (Figure 7A). In the TNF protein, the RMSD of the original ligand was 2.375 Å. The hydrogen bonding between the pro-ligand A7M and the 6OOY protein acted on the amino acid residue with SER-60 (2.9Å), TYS-151(1.8Å), and the free binding energy with protein was -4.87 kcal/mol. Calycosin formed hydrogen bond with amino acid residue TYS-151 (2.2Å), and the free binding energy with protein is -6.7 kcal/mol (Figure 7B).

## DISCUSSION

Sum-up of network pharmacology-based data, our bioinformatics findings using systematic pharmacology revealed the candidate and vital biotargets, biological functions, molecular pathways of calycosin-anti-meningitis, respectively. More significantly, all vital biotargets of calycosin-anti-meningitis were eventually identified, including EGFR, TNF, EGF, ATM, ESR1, CASP8, NGF. Molecular docking results demonstrated that the binding capacity of calycosin with meningitis was significant, indicating the potential and pharmacological activities of calycosin against meningitis. Reportedly, EGFR, a transmembrane glycoprotein, can biologically activate some key signaling pathways response to extracellular irritation, such as NF-kappa-B signaling pathway [14]. Another evidence show that transactivation of EGFR may contribute to the development of bacterial meningitis-induced neuroinflammation [15]. TNF, a proinflammatory family, can be activated in intracephalic inflammation based on cytokine stress induced by pneumococcal meningitis, such as TNF-α and IL-1β [16]. EGF, a functional hormone, may promote the differentiation of certain epidermal and epithelial tissues and proliferation of several fibroblasts [17]. ATM, a nuclear phosphoprotein, may function as a DNA-damage sensor for screening genotoxic stresses and cell death [18]. ESR1, an estrogen receptor alpha, is linked to the modulation of specific gene expression that is contribute to control cell proliferation and differentiation in the tissue [19]. CASP8, a cysteine protease, mediates the activation of proteases that are responsible for the TNF signal-induced cell apoptosis or death [20]. NGF, a nerve growth hormone, exerts the key roles in the maintenance and enhancement of nervous systems for inducing neuronal proliferation, differentiation and survival [21]. As reported in some studies, NGF may prevent memory impairment and re-induce hippocampal neurotrophin expression of pneumococcal meningitis in rats [22]. However, so far, there are no scientific reports regarding the associations between EGF, ATM, CASP8 and meningitis. Together with current bioinformatics data and reference reviews, these network pharmacology-based findings revealed all candidate biological targets, processes and pharmacological pathways of calycosin-anti- meningitis, characterized with specific inhibition of cytonecrosis- and inflammation-associated signaling pathways exerted by calycosin. Interestingly, calycosin may be a potential active ingredient to treat meningitis before further experimental validation.

## CONCLUSIONS

In summary, the bioinformatics approach using network pharmacology can be used to effectively uncover the candidate and vital biotargets, functional processes, molecular signals of calycosin-anti-meningitis. Markedly, all vital biotargets of calycosin-anti-meningitis are identified, and at least some of new anti-meningitis targets may include EGF, ATM, CASP8.

## MATERIALS AND METHODS

### Preparation of genes/targets of calycosin-anti-meningitis

All candidate genes/targets of calycosin were screened out and collected by use of traditional Chinese medicine systems pharmacology (TCMSP), Swiss Target Prediction, SuperPred-based databases, and all meningitis-associated genes/targets were gained through the databases of DisGeNET, Genecards. All the collected genes/targets of calycosin and meningitis were merged before being rectified by Uniprot database. After being mapped using Funrich software, the anti-meningitis targets exerted of calycosin were identified, as described previously [23, 24].

### Identifying the vital target and interactive network

The candidate targets of calycosin-anti-meningitis were further assayed for harvesting target-functional proteins through the functional protein association networks (STRING) database based on minimum interaction value. By using the Cytoscape software, the identified targets of calycosin-anti-meningitis were used to establish a protein-protein interaction (PPI) network. To further use the NetworkAnalyzer setting for topological parameters, the vital targets of calycosin-anti-meningitis were screened, followed by visualization of the vital targets [25, 26].

### Biological process and pathway enrichment analyses of vital targets

By using the Database for Annotation, Visualization and Integrated Discovery (DAVID) database, all biological processes and Kyoto Encyclopedia of Genes and Genomes (KEGG) pathways of the vital targets of calycosin-anti-meningitis were revealed and visualized respectively. And then the targets were re-assayed by Cytoscape to form an interaction network of disease-drug-target-pathway. By use of the Omicshare tool according to -LogP value, the advanced bubble diagrams of biological processes and KEGG signaling pathways were revealed and highlighted [27, 28].

### Molecular docking assay

Using chemical-protein binding method, the vital targets of EGFR, TNF were screened out and identified for calycosin-based molecular docking analysis. After searching for EGFR and TNF specific proteins through the PDB database, 5UGC, 6OOY ligands were selected to dock with the calycosin compound. The ChemBio3D Draw in Chem Bio Office 2010 software was used to conduct three-dimensional structure of calycosin before docking the molecular structures of EGFR, TNF following MGLTools in Autodock Vina software. The rationality of the docking parameter setting was assessed according to the root mean square deviation (RMSD) of the ligand molecule. And the RMSD ≤ 4 Å was the threshold for the conformation of the ligand molecule [29, 30].

## Supplementary Material

Supplementary Figure 1

Supplementary Table 1
