# Supplementary material for: Pharmacological targets and mechanisms of calycosin against meningitis
Source: Aging (Albany NY). 2020 Oct 8;12(19):19468–92. doi: 10.18632/aging.103886 (PMC7732281; doi:10.18632/aging.103886)
Supplement: Supplementary Figure 1 [file aging-12-103886-s001..pdf]

SUPPLEMENTARY FIGURE

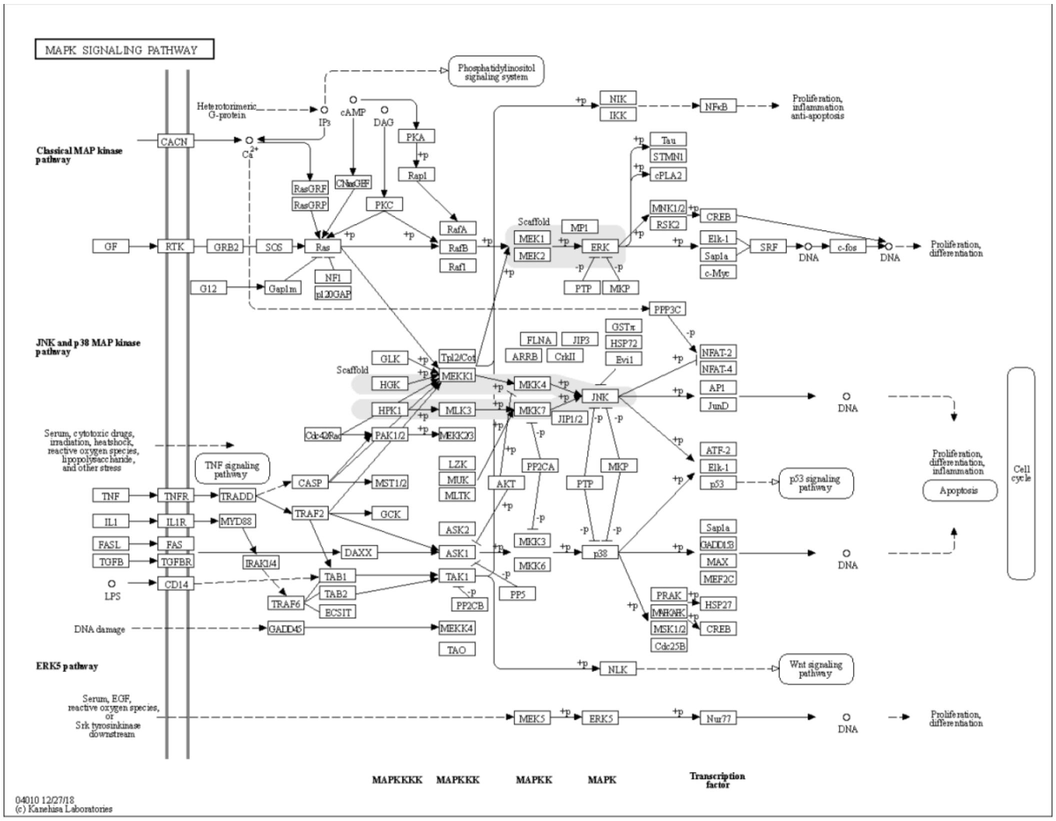

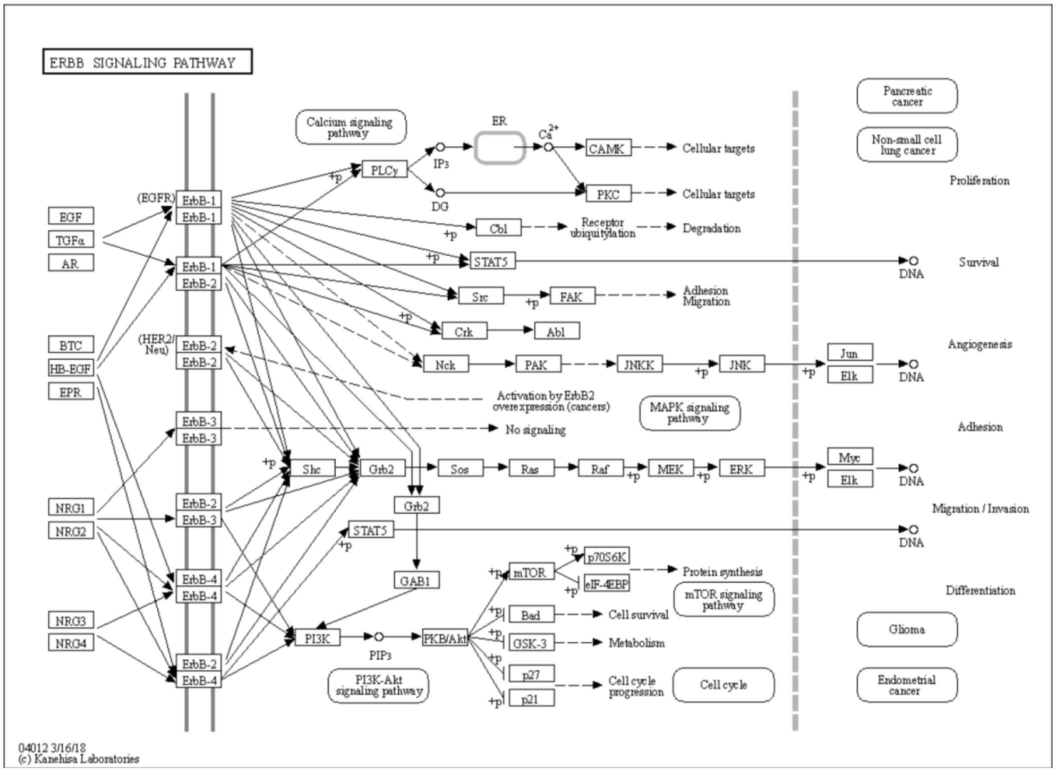

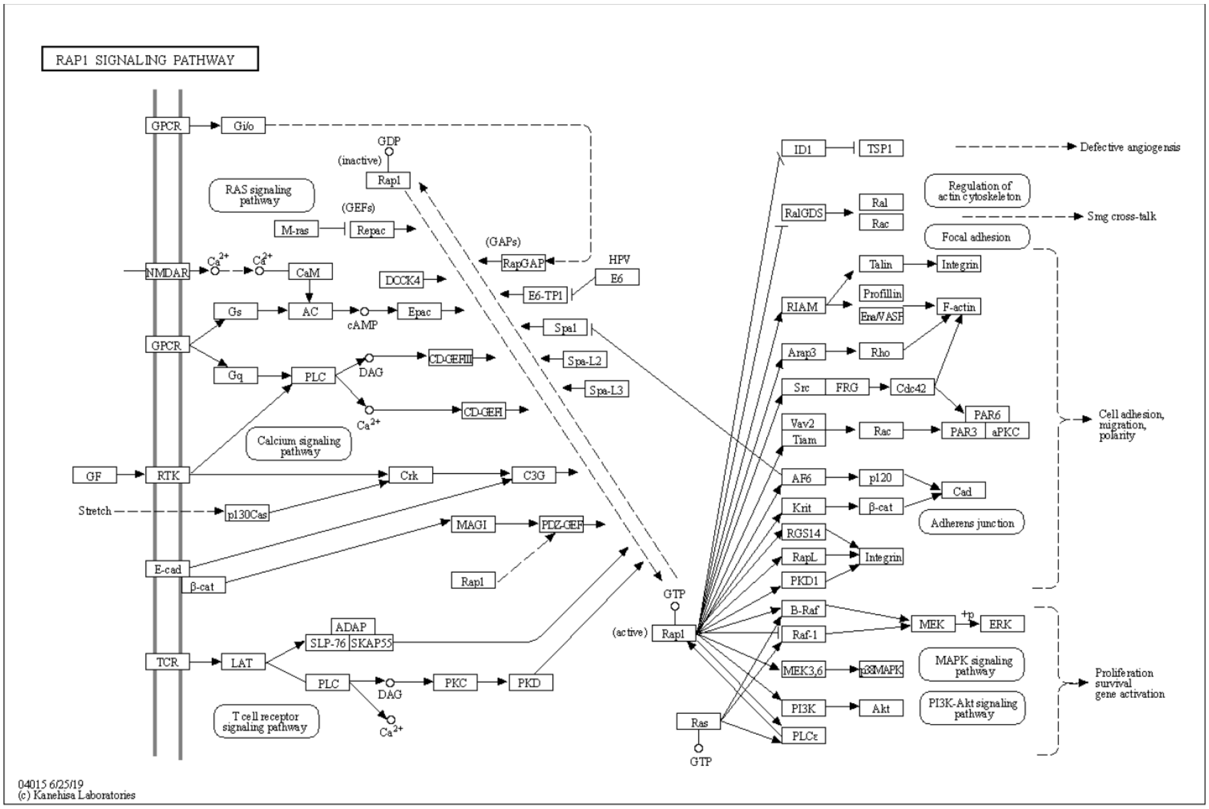

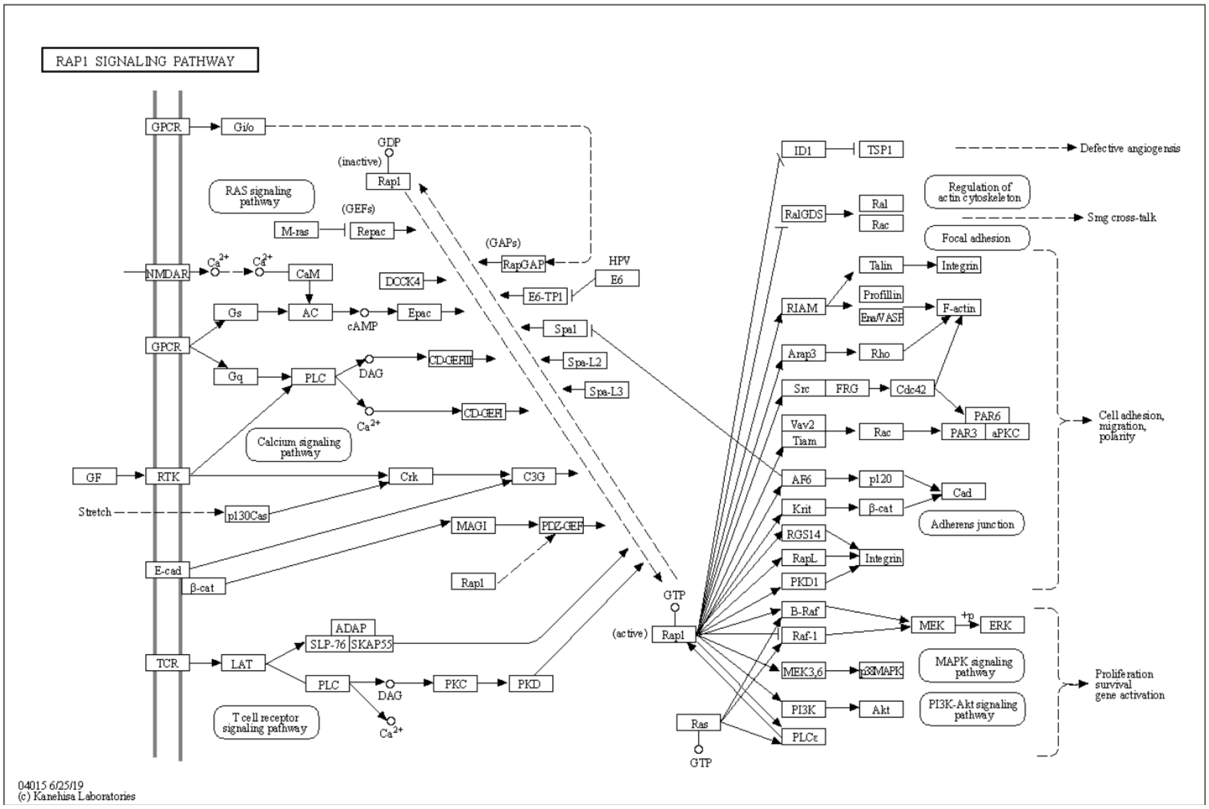
RAP1 SIGNALING PATHWAY
04015 6/25/19
(c) Kanehisa Laboratories

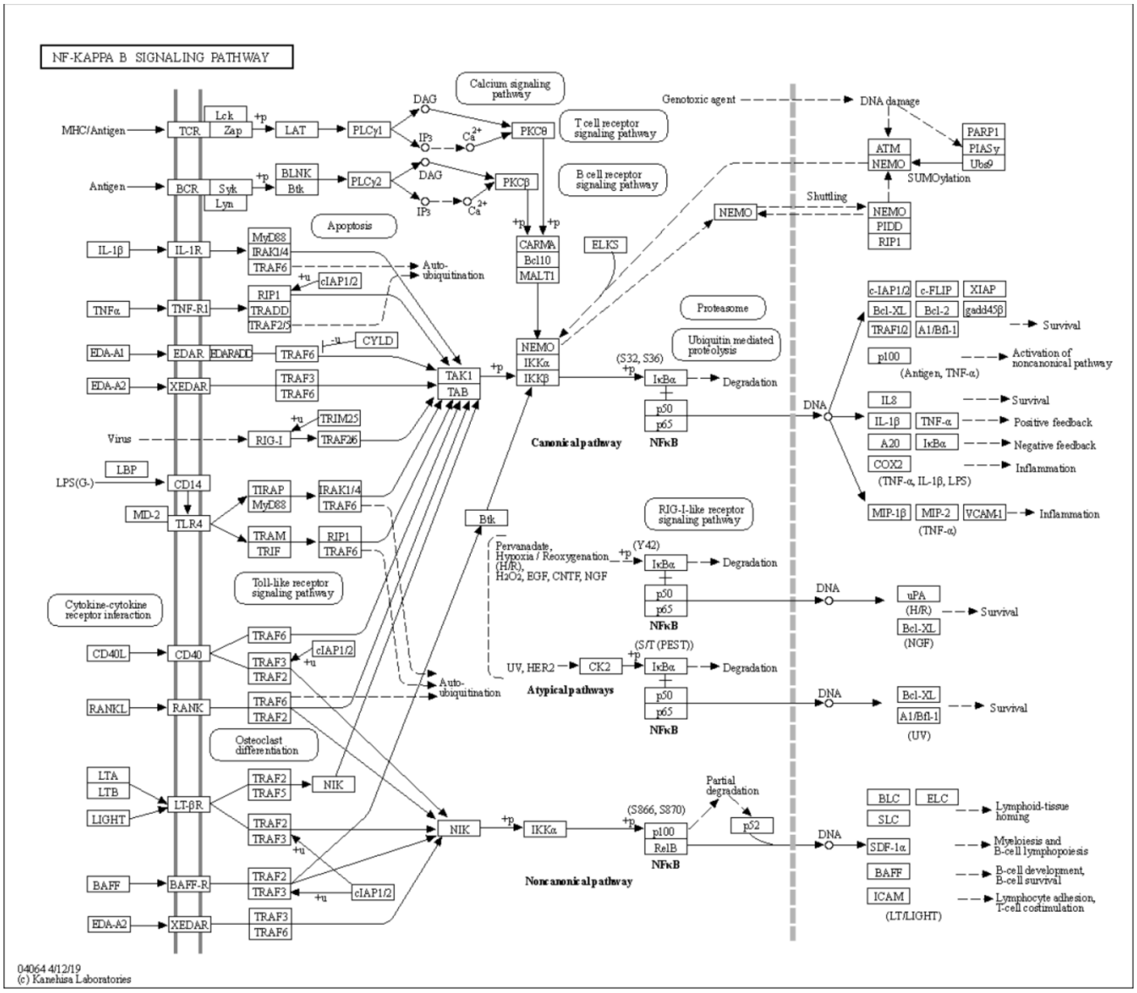
NF-KAPPA B SIGNALING PATHWAY
Calcium signaling pathway
T cell receptor signaling pathway
B cell receptor signaling pathway
Genotoxic agent
DNA damage
MHC/Antigen
Antigen
IL-1β
TNFα
EDA-A1
EDA-A2
Virus
LPS(G-)
Apoptosis
Proteasome
Ubiquitin mediated proteolysis
Canonical pathway
Atypical pathways
Noncanonical pathway
Toll-like receptor signaling pathway
RIG-I-like receptor signaling pathway
Cytokine-cytokine receptor interaction
Osteoclast differentiation
Survival
Activation of noncanonical pathway
Positive feedback
Negative feedback
Inflammation
Degradation
Partial degradation
Lymphoid-tissue homing
Myeloiesis and B-cell lymphopoiesis
B-cell development, B-cell survival
Lymphocyte adhesion, T-cell costimulation
04064 4/12/19
(c) Kanehisa Laboratories

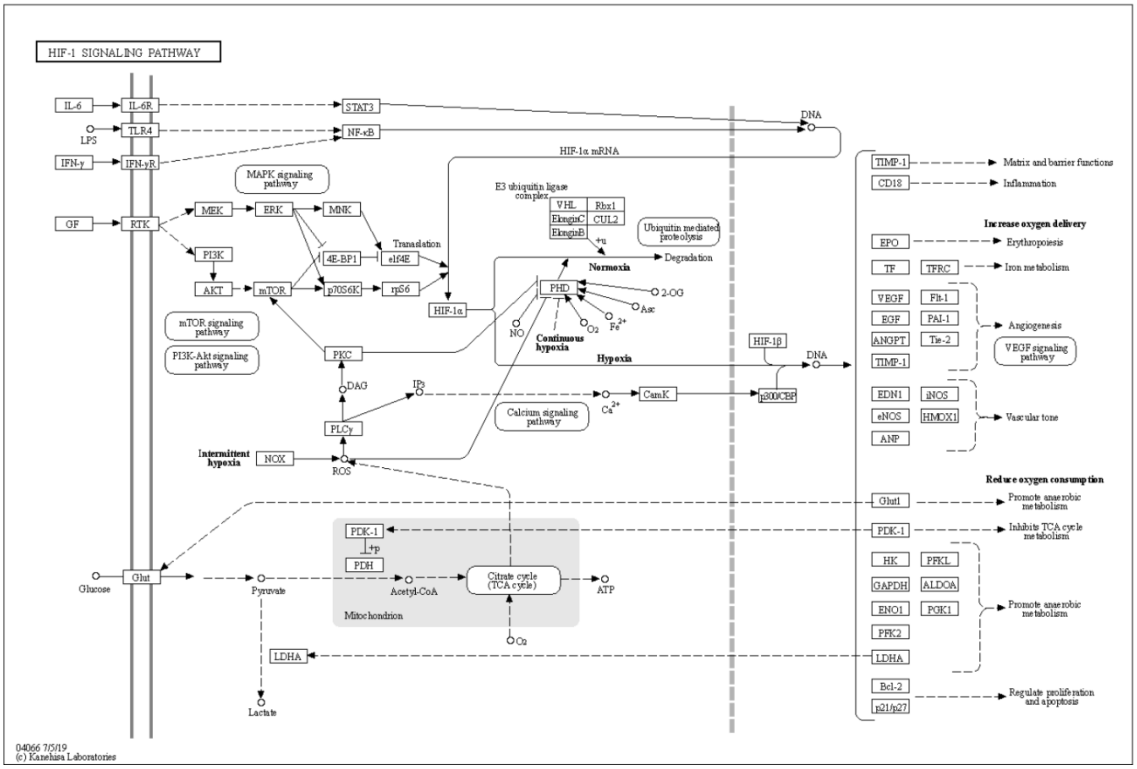
HIF-1 SIGNALING PATHWAY
MAPK signaling pathway
mTOR signaling pathway
PI3K-Akt signaling pathway
Calcium signaling pathway
VEGF signaling pathway
E3 ubiquitin ligase complex
Ubiquitin mediated proteolysis
Normoxia
Hypoxia
Continuous hypoxia
Intermittent hypoxia
HIF-1α mRNA
Translation
Degradation
Mitochondrion
Citrate cycle (TCA cycle)
Glucose
Pyruvate
Acetyl-CoA
Lactate
ATP
Matrix and barrier functions
Inflammation
Increase oxygen delivery
Erythropoiesis
Iron metabolism
Angiogenesis
Vascular tone
Reduce oxygen consumption
Promote anaerobic metabolism
Inhibits TCA cycle metabolism
Regulate proliferation and apoptosis
04066 7/5/19
(c) Kanehisa Laboratories

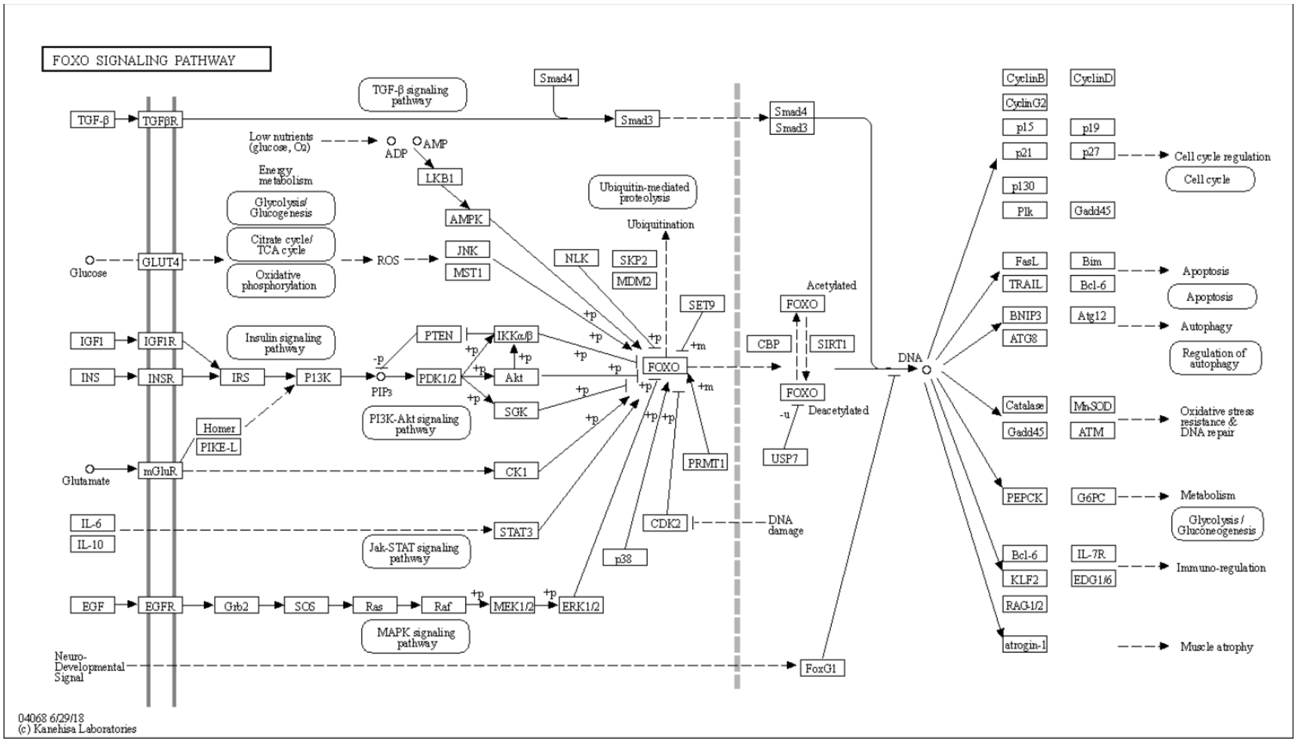
FOXO SIGNALING PATHWAY
TGF-β
TGFβR
TGF-β signaling pathway
Smad4
Smad3
Smad4
Smad3
Low nutrients (glucose, O2)
ADP
AMP
LKB1
Energy metabolism
Glycolysis/ Glucogenesis
Citrate cycle/ TCA cycle
Oxidative phosphorylation
AMPK
Ubiquitin-mediated proteolysis
Ubiquitination
Glucose
GLUT4
ROS
JNK
MST1
NLK
SKP2
MDM2
SET9
Acetylated
FOXO
CBP
SIRT1
FOXO
Deacetylated
USP7
IGF1
IGF1R
Insulin signaling pathway
PTEN
IKKα/β
INS
INSR
IRS
PI3K
PIP3
PDK1/2
Akt
SGK
FOXO
DNA
PI3K-Akt signaling pathway
Homer
PIKE-L
Glutamate
mGluR
CK1
PRMT1
IL-6
IL-10
STAT3
Jak-STAT signaling pathway
CDK2
DNA damage
p38
EGF
EGFR
Grb2
SOS
Ras
Raf
MEK1/2
ERK1/2
MAPK signaling pathway
Neuro-Developmental Signal
FoxG1
CyclinB
CyclinD
CyclinG2
p15
p19
p21
p27
Cell cycle regulation
Cell cycle
p130
Plk
Gadd45
FasL
Bim
Apoptosis
TRAIL
Bcl-6
Apoptosis
BNIP3
Atg12
Autophagy
ATG8
Regulation of autophagy
Catalase
MnSOD
Oxidative stress resistance & DNA repair
Gadd45
ATM
PEPCK
G6PC
Metabolism
Glycolysis / Gluconeogenesis
Bcl-6
IL-7R
Immuno-regulation
KLF2
EDG1/6
RAG-1/2
atrogin-1
Muscle atrophy
04068 6/29/18
(c) Kanehisa Laboratories

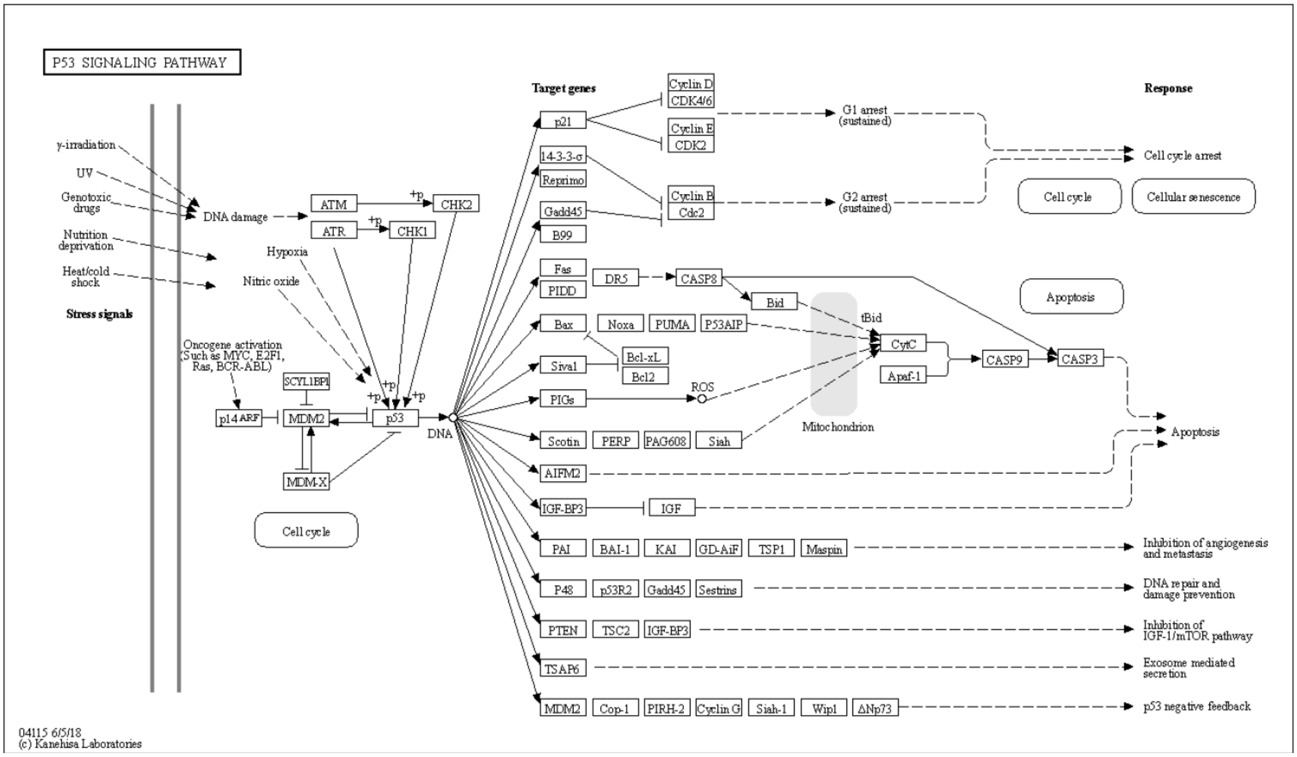
P53 SIGNALING PATHWAY
γ-irradiation
UV
Genotoxic drugs
Nutrition deprivation
Heat/cold shock
Stress signals
DNA damage
ATM
ATR
CHK2
CHK1
Hypoxia
Nitric oxide
Oncogene activation (Such as MYC, E2F1, Ras, BCR-ABL)
SCYL1BP1
p14 ARF
MDM2
p53
DNA
MDM-X
Cell cycle
Target genes
p21
Cyclin D
CDK4/6
Cyclin E
CDK2
14-3-3-σ
Reprimo
Gadd45
Cyclin B
Cdc2
B99
G1 arrest (sustained)
G2 arrest (sustained)
Response
Cell cycle arrest
Cell cycle
Cellular senescence
Fas
PIDD
DR5
CASP8
Bid
tBid
Bax
Noxa
PUMA
P53AIP
CytC
Apaf-1
CASP9
CASP3
Apoptosis
Sival
Bcl-xL
Bcl2
PIGs
ROS
Mitochondrion
Scotin
PERP
PAG608
Siah
Apoptosis
AIFM2
IGF-BP3
IGF
PAI
BAI-1
KAI
GD-AiF
TSP1
Maspin
Inhibition of angiogenesis and metastasis
P48
p53R2
Gadd45
Sestrins
DNA repair and damage prevention
PTEN
TSC2
IGF-BP3
Inhibition of IGF-1/mTOR pathway
TSAP6
Exosome mediated secretion
MDM2
Cop-1
PIRH-2
Cyclin G
Siah-1
Wip1
ΔNp73
p53 negative feedback
04115 6/5/18
(c) Kanehisa Laboratories

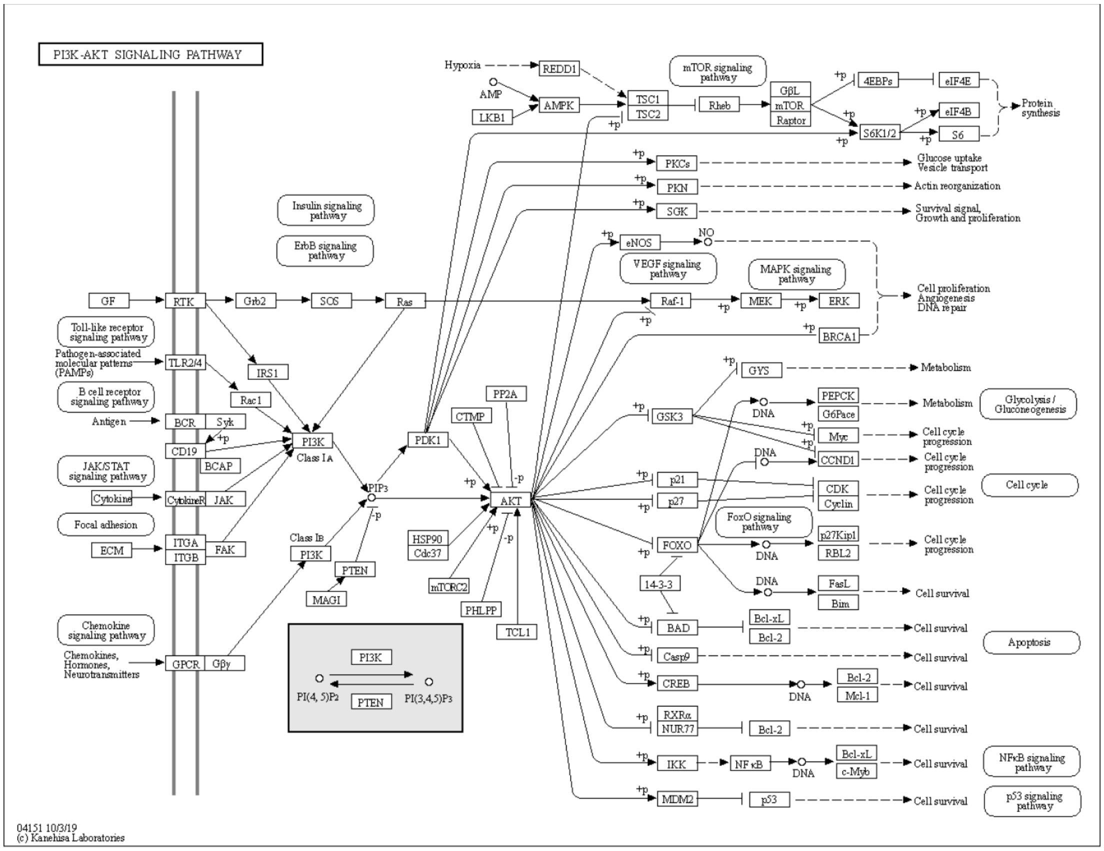
PI3K-AKT SIGNALING PATHWAY
Hypoxia
REDD1
AMP
AMPK
LKB1
TSC1
TSC2
Rheb
mTOR signaling pathway
GβL
mTOR
Raptor
4EBPs
eIF4E
eIF4B
S6K1/2
S6
Protein synthesis
PKCs
Glucose uptake
Vesicle transport
PKN
Actin reorganization
SGK
Survival signal, Growth and proliferation
Insulin signaling pathway
ErbB signaling pathway
eNOS
NO
VEGF signaling pathway
MAPK signaling pathway
GF
RTK
Grb2
SOS
Ras
Raf-1
MEK
ERK
Cell proliferation
Angiogenesis
DNA repair
BRCA1
Toll-like receptor signaling pathway
Pathogen-associated molecular patterns (PAMPs)
TLR2/4
IRS1
Rac1
B cell receptor signaling pathway
Antigen
BCR
Syk
CD19
BCAP
PI3K
Class IA
JAK/STAT signaling pathway
Cytokine
CytokineR
JAK
Focal adhesion
ECM
ITGA
ITGB
FAK
Chemokine signaling pathway
Chemokines, Hormones, Neurotransmitters
GPCR
Gβγ
Class IB
PIP3
PTEN
MAGI
PDK1
PP2A
CTMP
AKT
HSP90
Cdc37
mTORC2
PHLPP
TCL1
PI(4,5)P2
PI(3,4,5)P3
GYS
Metabolism
GSK3
DNA
PEPCK
G6Pace
Glycolysis / Gluconeogenesis
Myc
CCND1
Cell cycle progression
p21
p27
CDK
Cyclin
Cell cycle
FoxO signaling pathway
FOXO
p27Kip1
RBL2
14-3-3
FasL
Bim
Cell survival
BAD
Bcl-xL
Bcl-2
Apoptosis
Casp9
CREB
Mcl-1
RXRα
NUR77
IKK
NFκB
c-Myb
NFκB signaling pathway
MDM2
p53
p53 signaling pathway
04151 10/3/19
(c) Kanehisa Laboratories

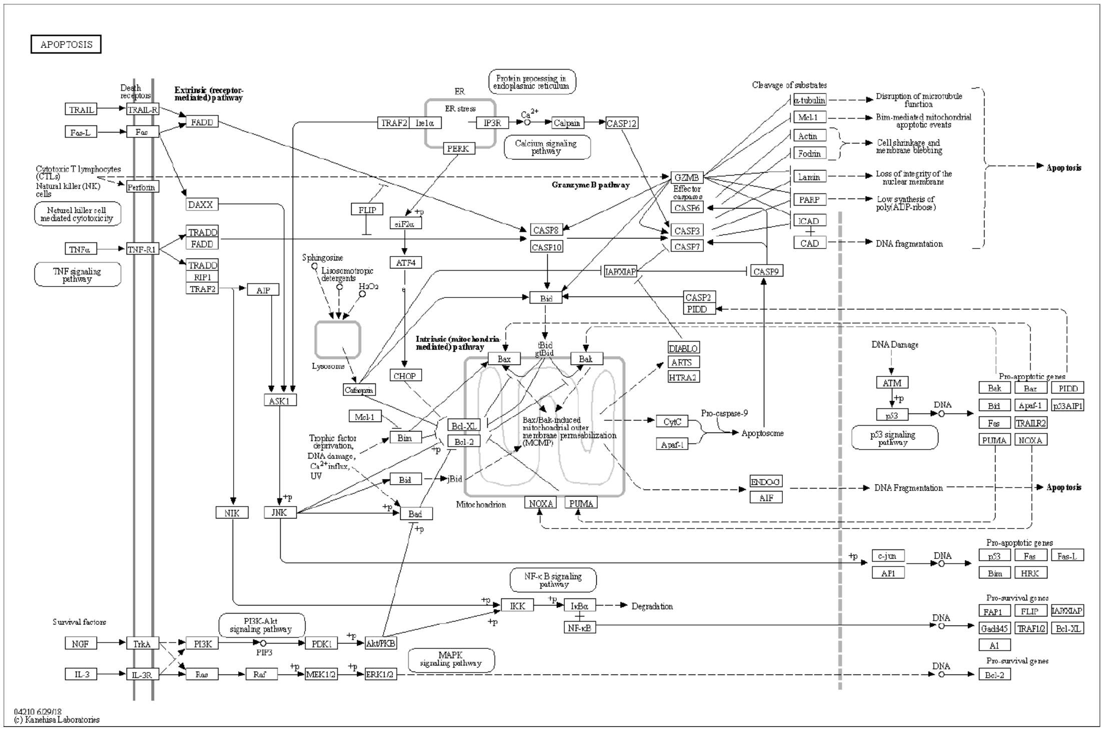
APOPTOSIS
Death receptors
Extrinsic (receptor-mediated) pathway
TRAIL
TRAIL-R
Fas-L
Fas
FADD
Cytotoxic T lymphocytes (CTLs)
Natural killer (NK) cells
Perforin
Natural killer cell mediated cytotoxicity
DAXX
TNFα
TNF-R1
TRADD
TNF signaling pathway
RIP1
TRAF2
AIP
ER
ER stress
Protein processing in endoplasmic reticulum
Ire1α
IP3R
Ca2+
Calpain
CASP12
PERK
Calcium signaling pathway
FLIP
eIF2α
ATF4
CHOP
Granzyme B pathway
GZMB
Effector caspases
CASP6
CASP3
CASP7
CASP8
CASP10
IAP/XIAP
CASP9
Sphingosine
Lysosomotropic detergents
H2O2
Lysosome
Cathepsin
ASK1
CASP2
PIDD
Bid
tBid
Intrinsic (mitochondria mediated) pathway
Bax
Bak
DIABLO
ARTS
HTRA2
Mcl-1
Bcl-XL
Bcl-2
Bim
Bad
Trophic factor deprivation, DNA damage, Ca2+ influx, UV
Bax/Bak-induced mitochondrial outer membrane permeabilization (MOMP)
CytC
Apaf-1
Pro-caspase-9
Apoptosome
ENDOG
AIF
DNA Fragmentation
Mitochondrion
NOXA
PUMA
NIK
JNK
Cleavage of substrates
α-tubulin
Disruption of microtubule function
Bim-mediated mitochondrial apoptotic events
Actin
Fodrin
Cell shrinkage and membrane blebbing
Lamin
Loss of integrity of the nuclear membrane
PARP
Low synthesis of poly(ADP-ribose)
ICAD
CAD
DNA fragmentation
Apoptosis
DNA Damage
ATM
p53
p53 signaling pathway
DNA
Pro-apoptotic genes
PIDD
Apaf-1
p53AIP1
TRAILR2
c-jun
AP1
HRK
NF-κB signaling pathway
IKK
IκBα
NF-κB
Degradation
Pro-survival genes
FLIP
IAP/XIAP
Gadd45
TRAF1/2
Bcl-XL
A1
Survival factors
NGF
TrkA
PI3K
PIP3
PDK1
PI3K-Akt signaling pathway
Akt/PKB
IL-3
IL-3R
Ras
Raf
MEK1/2
ERK1/2
MAPK signaling pathway
04210 6/29/18
(c) Kanehisa Laboratories

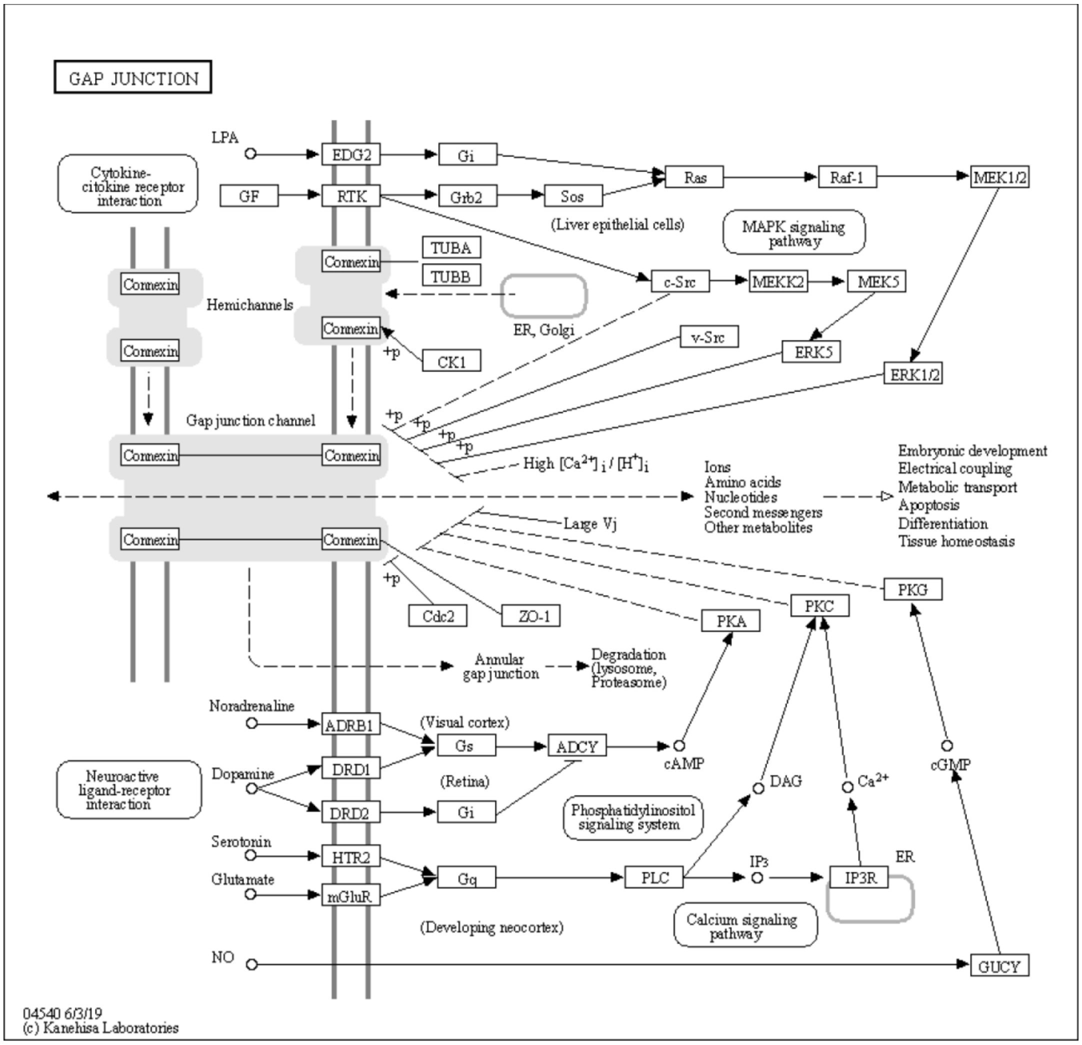
GAP JUNCTION
LPA
EDG2
Gi
Ras
Raf-1
MEK1/2
Cytokine-cytokine receptor interaction
GF
RTK
Grb2
Sos
(Liver epithelial cells)
MAPK signaling pathway
TUBA
TUBB
Connexin
c-Src
MEKK2
MEK5
Hemichannels
ER, Golgi
v-Src
ERK5
CK1
ERK1/2
+p
Gap junction channel
High [Ca2+]i / [H+]i
Ions
Amino acids
Nucleotides
Second messengers
Other metabolites
Embryonic development
Electrical coupling
Metabolic transport
Apoptosis
Differentiation
Tissue homeostasis
Large Vj
PKG
PKC
PKA
Cdc2
ZO-1
Annular gap junction
Degradation (lysosome, Proteasome)
Noradrenaline
ADRB1
(Visual cortex)
Gs
ADCY
cAMP
cGMP
Neuroactive ligand-receptor interaction
Dopamine
DRD1
(Retina)
DAG
Ca2+
DRD2
Phosphatidylinositol signaling system
Serotonin
HTR2
IP3
ER
Glutamate
mGluR
Gq
PLC
IP3R
(Developing neocortex)
Calcium signaling pathway
NO
GUCY
04540 6/3/19
(c) Kanehisa Laboratories

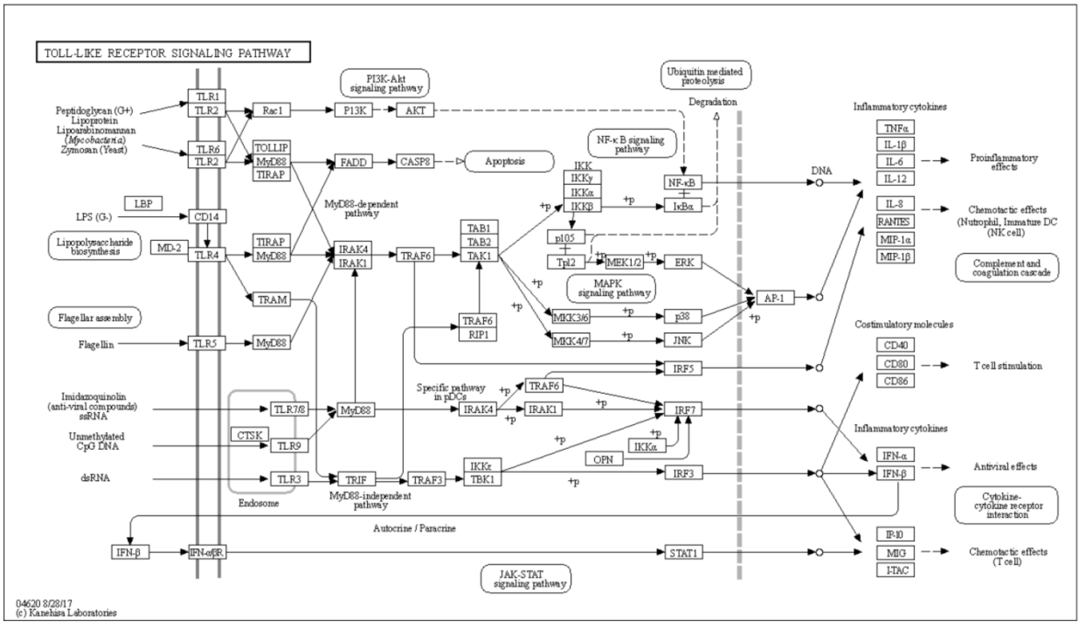
TOLL-LIKE RECEPTOR SIGNALING PATHWAY
PI3K-Akt signaling pathway
Ubiquitin mediated proteolysis
Degradation
Inflammatory cytokines
Peptidoglycan (G+)
Lipoprotein
Lipoarabinomannan (Mycobacteria)
Zymosan (Yeast)
TLR1
TLR2
TLR6
Rac1
P13K
AKT
TOLLIP
MyD88
TIRAP
FADD
CASP8
Apoptosis
NF-κB signaling pathway
IKK
IKKγ
IKKα
IKKβ
NF-κB
IκBα
DNA
TNFα
IL-1β
IL-6
IL-12
Proinflammatory effects
LBP
LPS (G-)
CD14
MyD88-dependent pathway
IL-8
RANTES
MIP-1α
MIP-1β
Chemotactic effects (Nutrophil, Immature DC (NK cell)
Lipopolysaccharide biosynthesis
MD-2
TLR4
IRAK4
IRAK1
TRAF6
TAB1
TAB2
TAK1
p105
Tpl2
MEK1/2
ERK
Complement and coagulation cascade
MAPK signaling pathway
AP-1
TRAM
Flagellar assembly
Flagellin
TLR5
RIP1
MKK3/6
MKK4/7
p38
JNK
Costimulatory molecules
CD40
CD80
CD86
T cell stimulation
IRF5
Imidazoquinolin (anti-viral compounds) ssRNA
TLR7/8
Specific pathway in pDCs
IRF7
Unmethylated CpG DNA
CTSK
TLR9
IKKα
OPN
Inflammatory cytokines
IFN-α
IFN-β
Antiviral effects
dsRNA
TLR3
TRIF
TRAF3
IKKε
TBK1
IRF3
Endosome
MyD88-independent pathway
Cytokine-cytokine receptor interaction
Autocrine / Paracrine
IFN-β
IFN-αβR
STAT1
IP-10
MIG
I-TAC
Chemotactic effects (T cell)
JAK-STAT signaling pathway
04620 8/28/17
(c) Kanehisa Laboratories

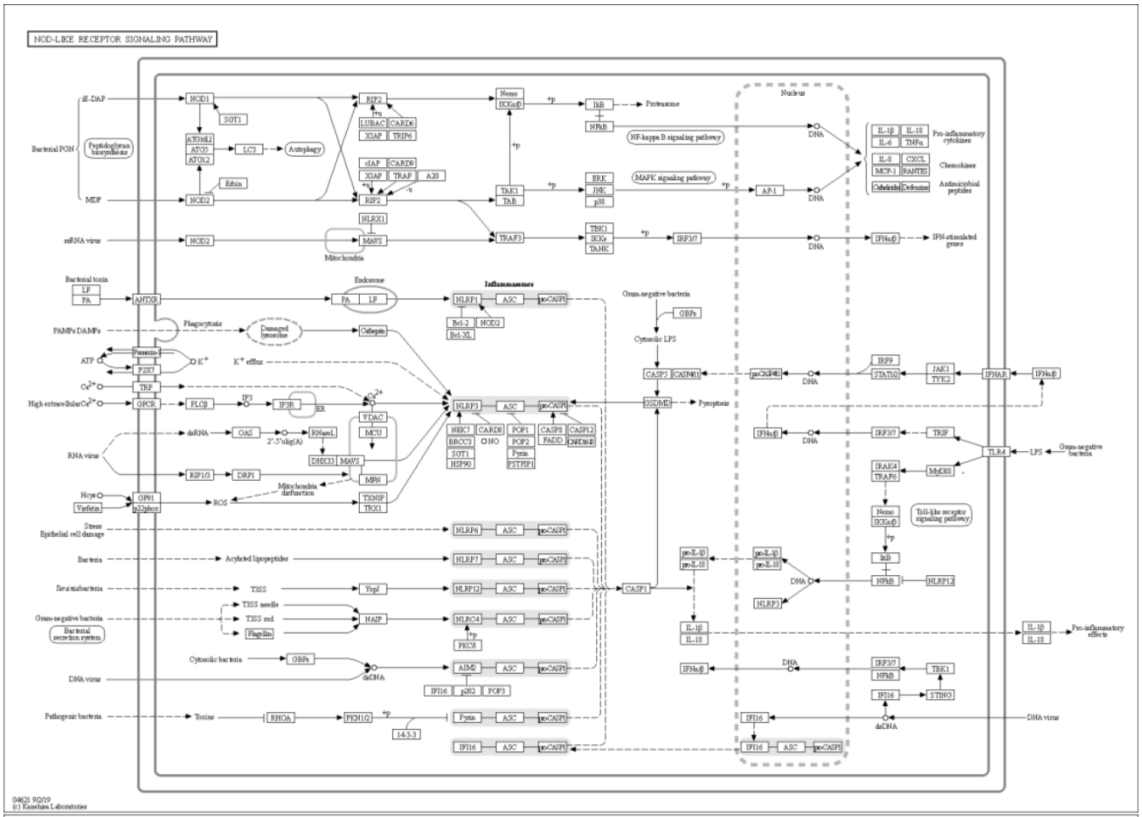
NOD-LIKE RECEPTOR SIGNALING PATHWAY
Nucleus
Inflammasomes
Pro-inflammatory cytokines
Chemokines
Antimicrobial peptides
Pyroptosis
(c) Kanehisa Laboratories

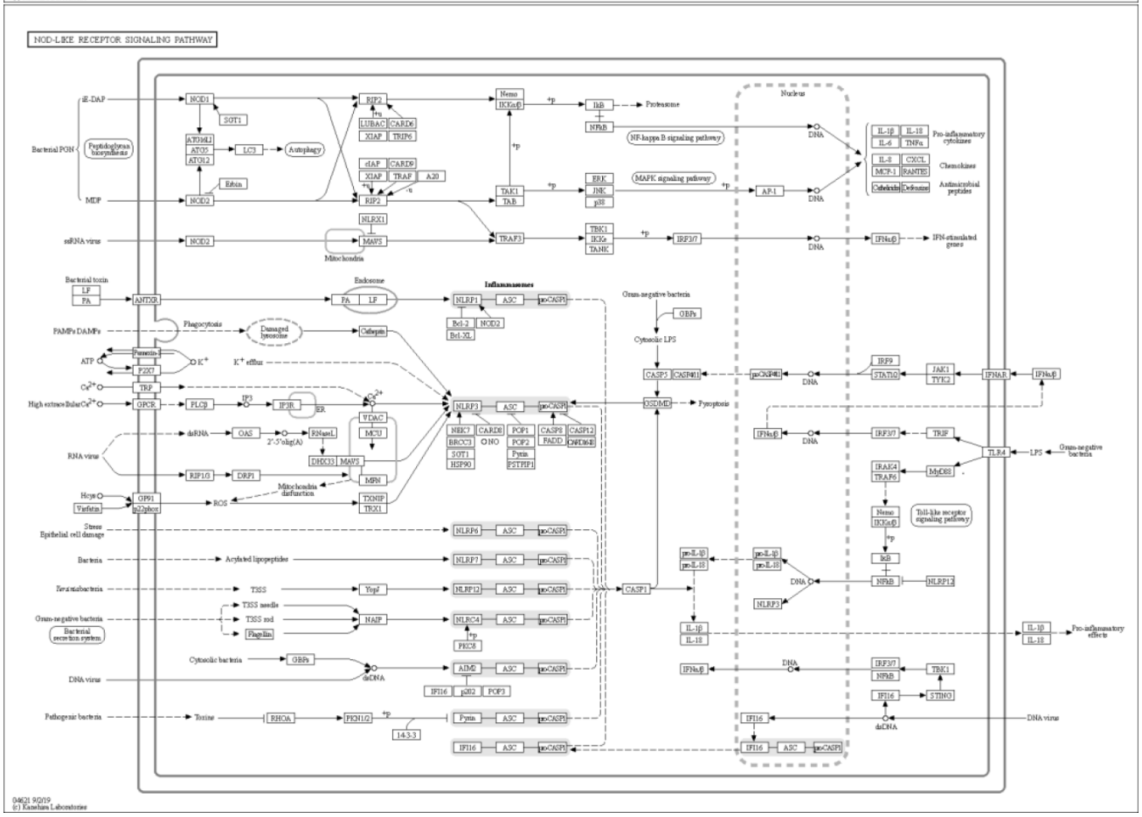
NOD-LIKE RECEPTOR SIGNALING PATHWAY
Nucleus
Inflammasomes
Pro-inflammatory cytokines
Chemokines
Antimicrobial peptides
Pyroptosis
(c) Kanehisa Laboratories

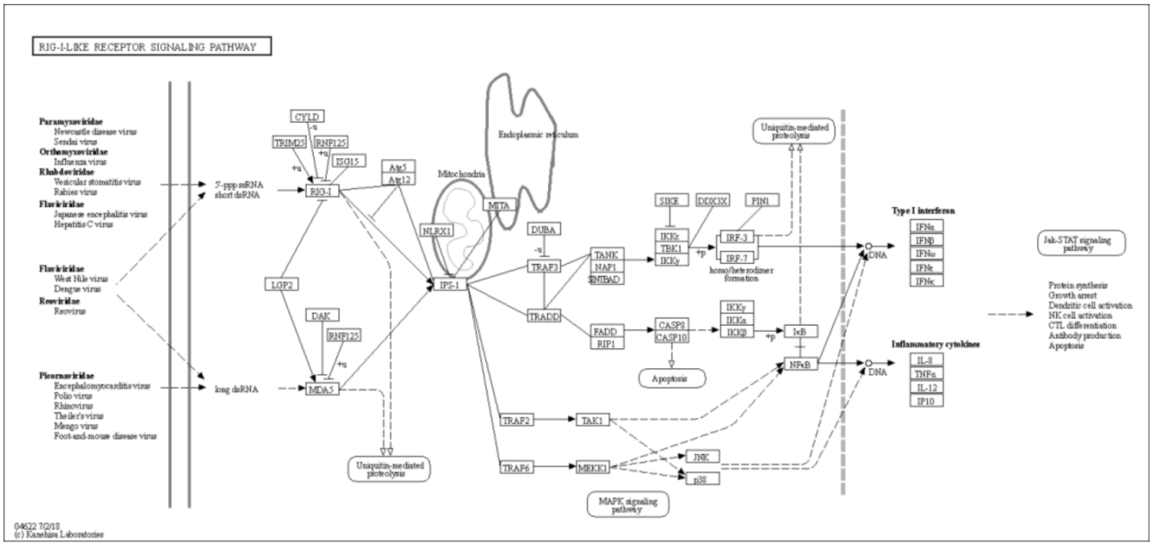

RIG-I-LIKE RECEPTOR SIGNALING PATHWAY
Paramyxoviridae
Newcastle disease virus
Sendai virus
Orthomyxoviridae
Influenza virus
Rhabdoviridae
Vesicular stomatitis virus
Rabies virus
Flaviviridae
Japanese encephalitis virus
Hepatitis C virus
Flaviviridae
West Nile virus
Dengue virus
Reoviridae
Reovirus
Picornaviridae
Encephalomyocarditis virus
Polio virus
Rhinovirus
Theiler's virus
Mengo virus
Foot-and-mouse disease virus
5'-ppp ssRNA
short dsRNA
long dsRNA
CYLD
TRIM25
RNF125
ISG15
RIG-I
Atg5
Atg12
LGP2
DAK
RNF125
MDA5
Mitochondria
Endoplasmic reticulum
NLRX1
MITA
IPS-1
DUBA
TRAF3
TANK
NAP1
SINTBAD
TRADD
FADD
RIP1
CASP8
CASP10
Apoptosis
SIKE
DDX3X
PIN1
IKKε
TBK1
IKKγ
IRF-3
IRF-7
homo/heterodimer formation
IKKγ
IKKα
IKKβ
IκB
NFκB
Uniquitin-mediated proteolysis
Uniquitin-mediated proteolysis
TRAF2
TAK1
TRAF6
MEKK1
JNK
p38
MAPK signaling pathway
DNA
DNA
Type I interferon
IFNα
IFNβ
IFNω
IFNε
IFNκ
Inflammatory cytokines
IL-8
TNFα
IL-12
IP10
Jak-STAT signaling pathway
Protein synthesis
Growth arrest
Dendritic cell activation
NK cell activation
CTL differentiation
Antibody production
Apoptosis
04622 7/2/18
(c) Kanehisa Laboratories

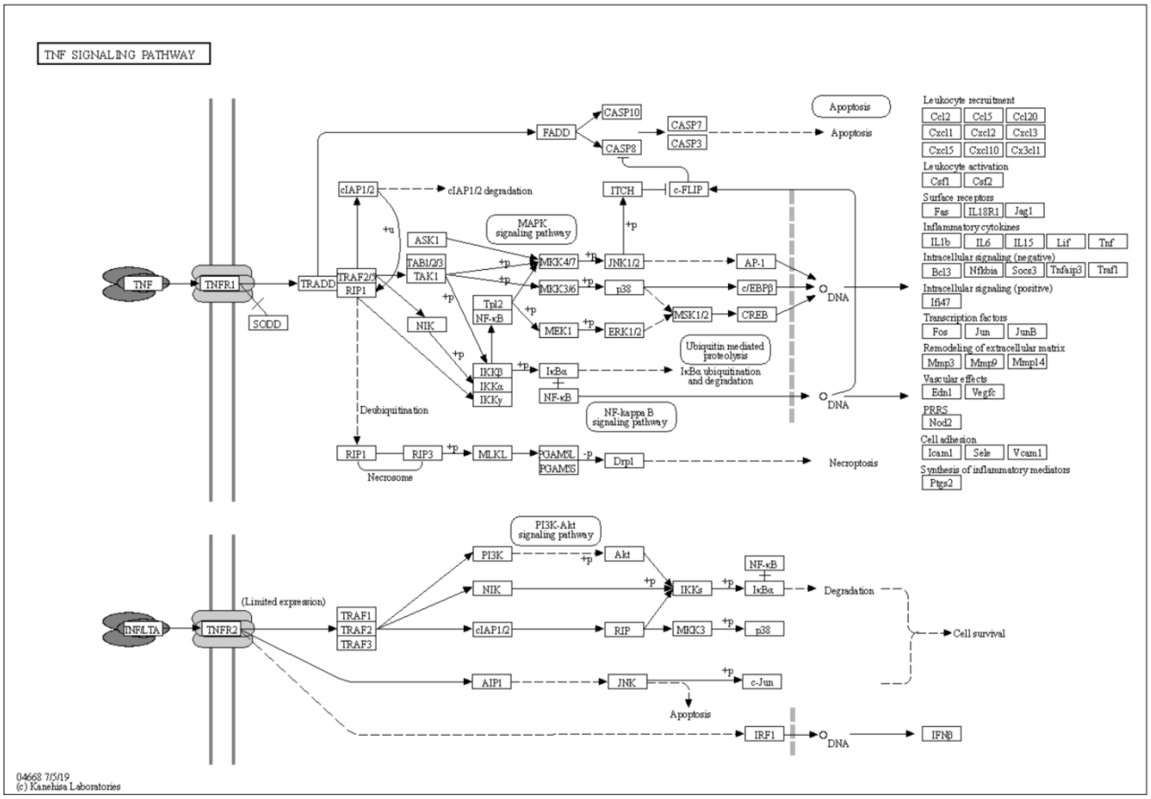

TNF SIGNALING PATHWAY
TNF
TNFR1
SODD
TRADD
TRAF2/5
RIP1
FADD
CASP10
CASP8
CASP7
CASP3
Apoptosis
Apoptosis
cIAP1/2
cIAP1/2 degradation
ITCH
c-FLIP
ASK1
TAB1/2/3
TAK1
MAPK signaling pathway
MKK4/7
MKK3/6
JNK1/2
p38
AP-1
c/EBPβ
MSK1/2
CREB
Tpl2
NF-κB
MEK1
ERK1/2
NIK
IKKβ
IKKα
IKKγ
IκBα
NF-κB
Ubiquitin mediated proteolysis
IκBα ubiquitination and degradation
NF-kappa B signaling pathway
DNA
DNA
Deubiquitination
RIP1
RIP3
MLKL
PGAM5L
PGAM5S
Drp1
Necroptosis
Necrosome
Leukocyte recruitment
Ccl2
Ccl5
Ccl20
Cxcl1
Cxcl2
Cxcl3
Cxcl5
Cxcl10
Cx3cl1
Leukocyte activation
Csf1
Csf2
Surface receptors
Fas
IL18R1
Jag1
Inflammatory cytokines
IL1b
IL6
IL15
Lif
Tnf
Intracellular signaling (negative)
Bcl3
Nfkbia
Socs3
Tnfaip3
Traf1
Intracellular signaling (positive)
Ifi47
Transcription factors
Fos
Jun
JunB
Remodeling of extracellular matrix
Mmp3
Mmp9
Mmp14
Vascular effects
Edn1
Vegfc
PRRS
Nod2
Cell adhesion
Icam1
Sele
Vcam1
Synthesis of inflammatory mediators
Ptgs2
TNF/LTA
TNFR2
(Limited expression)
TRAF1
TRAF2
TRAF3
PI3K-Akt signaling pathway
PI3K
Akt
NIK
IKKs
NF-κB
IκBα
Degradation
cIAP1/2
RIP
MKK3
p38
Cell survival
AIP1
JNK
c-Jun
Apoptosis
IRF1
DNA
IFNβ
04668 7/5/19
(c) Kanehisa Laboratories

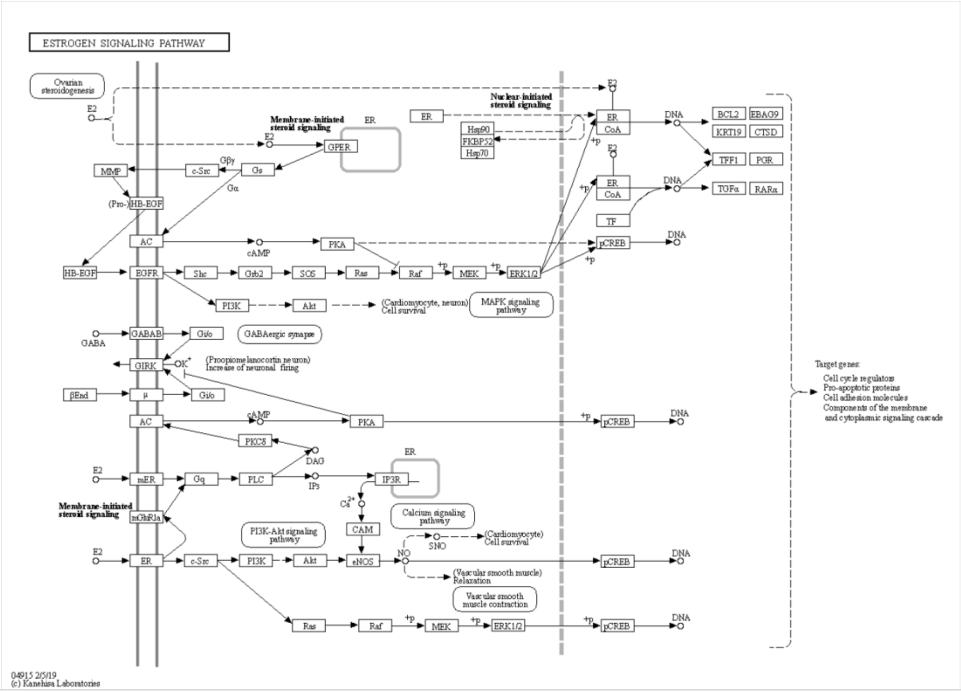

ESTROGEN SIGNALING PATHWAY
Ovarian steroidogenesis
E2
Membrane-initiated steroid signaling
Nuclear-initiated steroid signaling
ER
GPER
Hsp90
FKBP52
Hsp70
CoA
DNA
BCL2
EBAG9
KRT19
CTSD
TFF1
PGR
TGFα
RARα
TF
MMP
c-Src
Gs
Gβγ
Gα
(Pro-) HB-EGF
AC
cAMP
PKA
pCREB
HB-EGF
EGFR
Shc
Grb2
SOS
Ras
Raf
MEK
ERK1/2
+p
PI3K
Akt
(Cardiomyocyte, neuron) Cell survival
MAPK signaling pathway
GABA
GABAB
Gi/o
GABAergic synapse
GIRK
K+
(Proopiomelanocortin neuron) Increase of neuronal firing
βEnd
μ
PKCδ
DAG
mER
Gq
PLC
IP3
IP3R
Ca2+
mGluR1a
CAM
Calcium signaling pathway
PI3K-Akt signaling pathway
eNOS
NO
SNO
(Cardiomyocyte) Cell survival
(Vascular smooth muscle) Relaxation
Vascular smooth muscle contraction
Target genes:
Cell cycle regulators
Pro-apoptotic proteins
Cell adhesion molecules
Components of the membrane and cytoplasmic signaling cascade
04915 2/5/19
(c) Kanehisa Laboratories

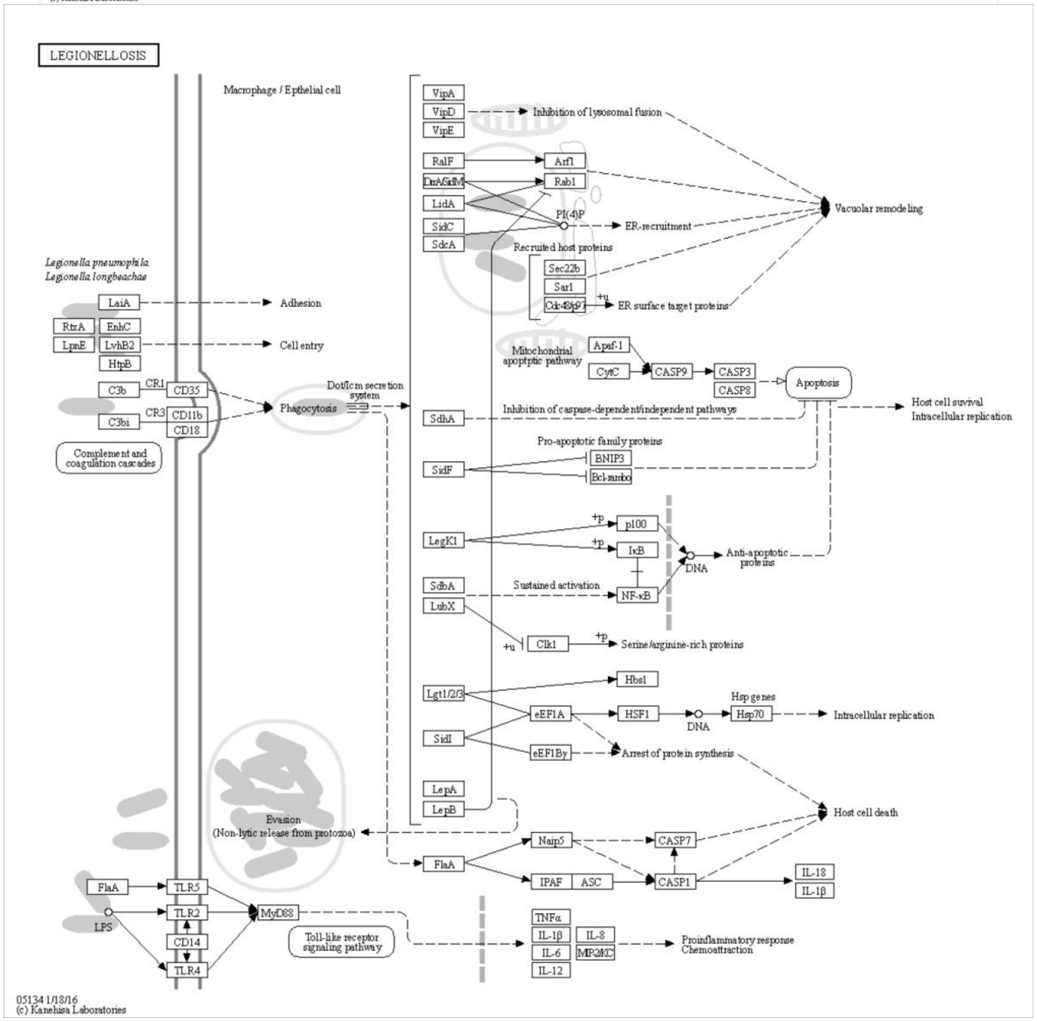

LEGIONELLOSIS
Macrophage / Epithelial cell
VipA
VipD
VipE
Inhibition of lysosomal fusion
RalF
Arf1
DrrA/SidM
Rab1
LidA
SidC
SdcA
PI(4)P
ER-recruitment
Vacuolar remodeling
Recruited host proteins
Sec22b
Sar1
Cdc48/p97
+u
ER surface target proteins
Legionella pneumophila
Legionella longbeachae
LaiA
Adhesion
RtxA
EnhC
LpnE
LvhB2
Cell entry
HtpB
C3b
CR1
CD35
C3bi
CR3
CD11b
CD18
Dot/Icm secretion system
Phagocytosis
Complement and coagulation cascades
Mitochondrial apoptotic pathway
Apaf-1
CytC
CASP9
CASP3
CASP8
Apoptosis
SdhA
Inhibition of caspase-dependent/independent pathways
Host cell survival
Intracellular replication
Pro-apoptotic family proteins
SidF
BNIP3
Bcl-rambo
LegK1
+p
p100
IκB
DNA
Anti-apoptotic proteins
SdbA
LubX
Sustained activation
NF-κB
Clk1
Serine/arginine-rich proteins
Hbs1
Lgt1/2/3
eEF1A
HSF1
Hsp genes
Hsp70
Intracellular replication
SidI
eEF1Bγ
Arrest of protein synthesis
LepA
LepB
Host cell death
Evasion
(Non-lytic release from protozoa)
FlaA
Naip5
CASP7
IPAF
ASC
CASP1
IL-18
IL-1β
TLR5
TLR2
LPS
CD14
TLR4
MyD88
Toll-like receptor signaling pathway
TNFα
IL-1β
IL-8
IL-6
MIP-2/KC
IL-12
Proinflammatory response
Chemoattraction
05134 1/18/16
(c) Kanehisa Laboratories

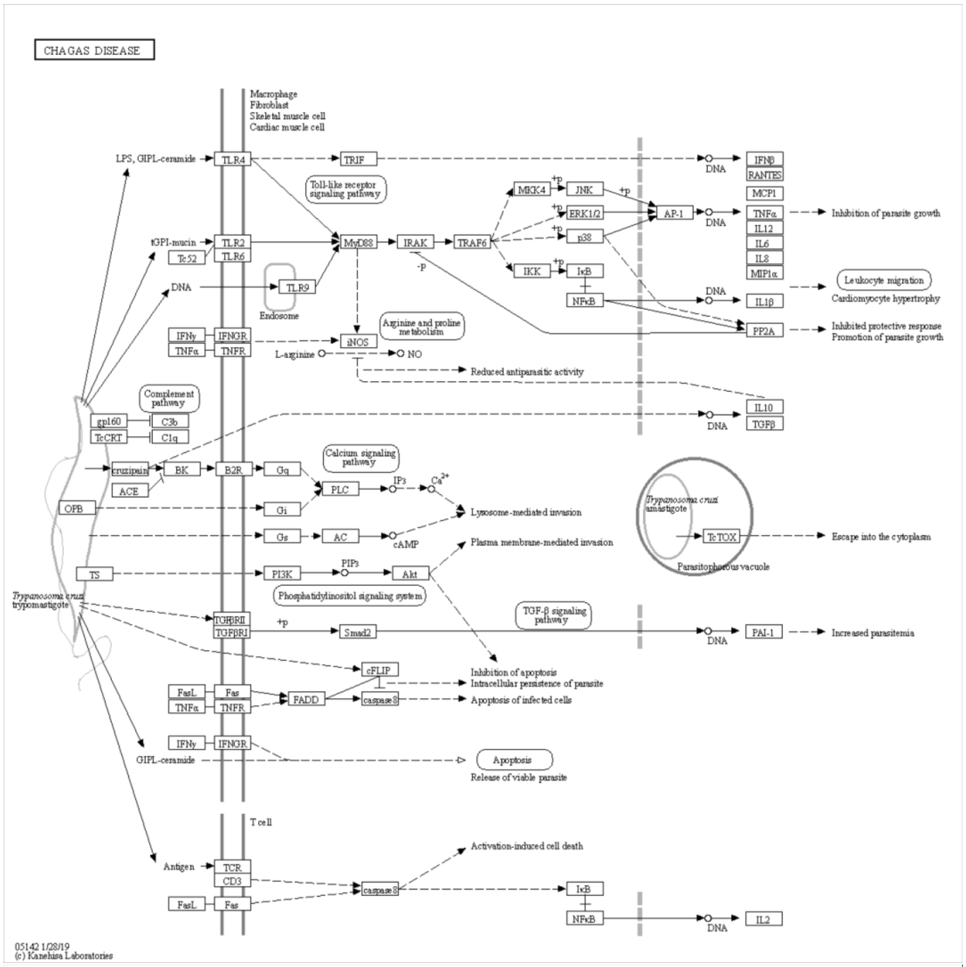

CHAGAS DISEASE
Macrophage
Fibroblast
Skeletal muscle cell
Cardiac muscle cell
LPS, GIPL-ceramide
TLR4
TRIF
DNA
IFNβ
RANTES
MCP1
TNFα
IL12
IL6
IL8
MIP1α
IL1β
PP2A
IL10
TGFβ
PAI-1
IL2
Toll-like receptor signaling pathway
MKK4
JNK
ERK1/2
p38
AP-1
IKK
IκB
NFκB
tGPI-mucin
Tc52
TLR2
TLR6
MyD88
IRAK
TRAF6
TLR9
Endosome
IFNγ
IFNGR
TNFα
TNFR
iNOS
L-arginine
NO
Arginine and proline metabolism
Reduced antiparasitic activity
Inhibition of parasite growth
Leukocyte migration
Cardiomyocyte hypertrophy
Inhibited protective response
Promotion of parasite growth
Complement pathway
gp160
C3b
TcCRT
C1q
cruzipain
BK
B2R
Gq
ACE
Calcium signaling pathway
PLC
IP3
Ca2+
OPB
Gi
Gs
AC
cAMP
Lysosome-mediated invasion
Plasma membrane-mediated invasion
TS
PI3K
PIP3
Akt
Phosphatidylinositol signaling system
Trypanosoma cruzi amastigote
TcTOX
Parasitophorous vacuole
Escape into the cytoplasm
Trypanosoma cruzi trypomastigote
TGFβRII
TGFβRI
Smad2
TGF-β signaling pathway
Increased parasitemia
cFLIP
FasL
Fas
FADD
caspase8
Inhibition of apoptosis
Intracellular persistence of parasite
Apoptosis of infected cells
GIPL-ceramide
Apoptosis
Release of viable parasite
T cell
Antigen
TCR
CD3
Activation-induced cell death
05142 1/28/19
(c) Kanehisa Laboratories

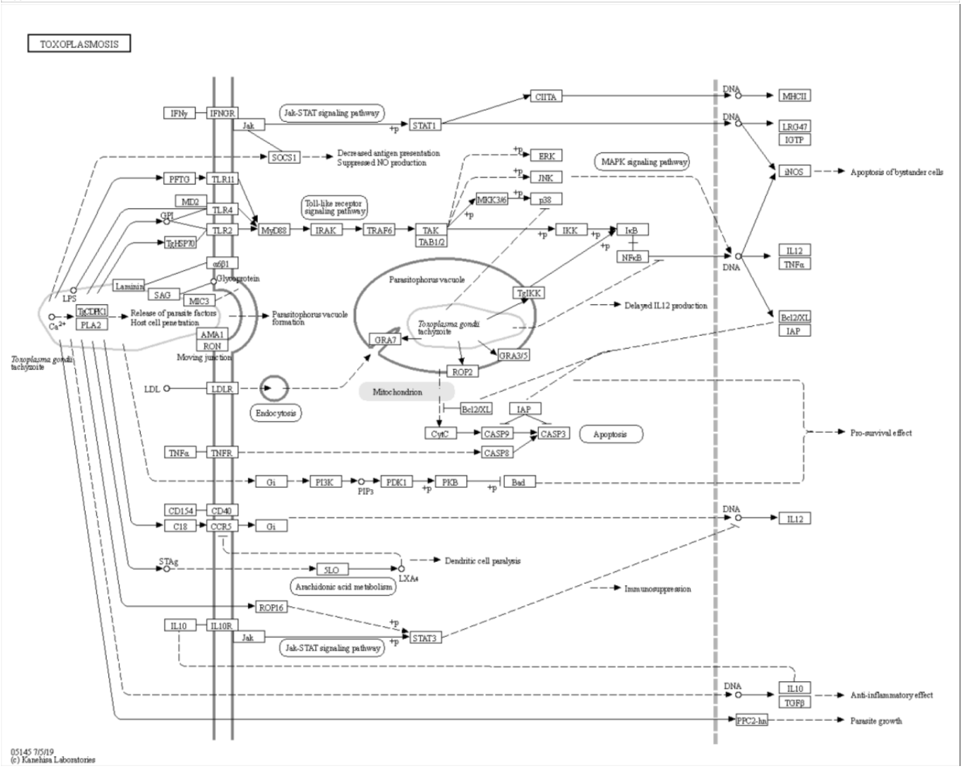

TOXOPLASMOSIS
IFNγ
IFNGR
Jak
Jak-STAT signaling pathway
STAT1
CIITA
DNA
MHCII
LRG47
IGTP
SOCS1
Decreased antigen presentation
Suppressed NO production
ERK
JNK
p38
MAPK signaling pathway
iNOS
Apoptosis of bystander cells
PFTG
TLR11
MD2
TLR4
GPI
TLR2
MyD88
IRAK
TRAF6
TAK
TAB1/2
MKK3/6
IKK
IκB
NFκB
Toll-like receptor signaling pathway
TgHSP70
IL12
TNFα
Laminin
SAG
MIC3
α6β1
Glycoprotein
LPS
Ca2+
TgCDPK1
PLA2
Release of parasite factors
Host cell penetration
Parasitophorus vacuole formation
AMA1
RON
Moving junction
Toxoplasma gondii tachyzoite
Parasitophorus vacuole
TgIKK
Delayed IL12 production
GRA7
GRA3/5
ROP2
Bcl2/XL
IAP
LDL
LDLR
Endocytosis
Mitochondrion
Bcl2/XL
CytC
CASP9
CASP3
Apoptosis
TNFR
CASP8
Pro-survival effect
Gi
PI3K
PIP3
PDK1
PKB
Bad
CD154
CD40
C18
CCR5
STAg
SLO
LXA4
Arachidonic acid metabolism
Dendritic cell paralysis
Immunosuppression
ROP16
IL10
IL10R
STAT3
Anti-inflammatory effect
TGFβ
PPC2-Ia
Parasite growth
05145 7/5/19
(c) Kanehisa Laboratories

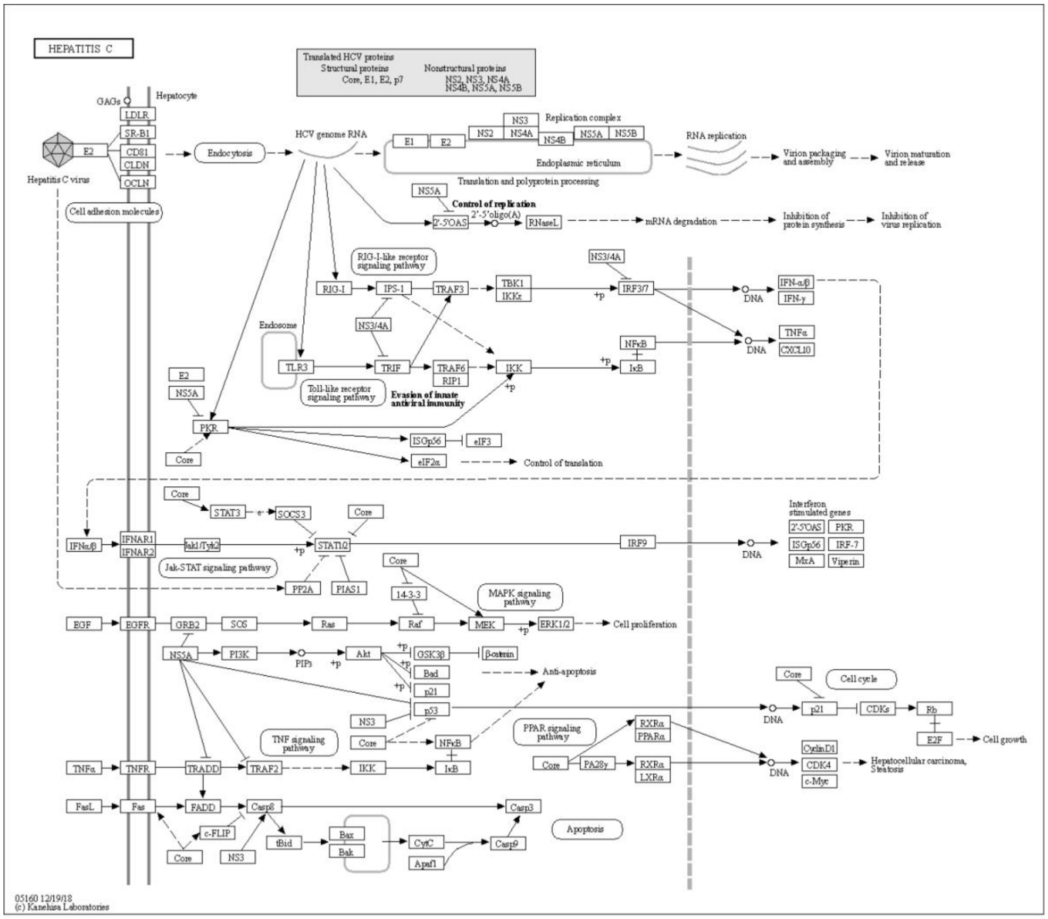
HEPATITIS C
Translated HCV proteins
Structural proteins
Core, E1, E2, p7
Nonstructural proteins
NS2, NS3, NS4A
NS4B, NS5A, NS5B
Hepatocyte
GAGs
LDLR
SR-B1
CD81
CLDN
OCLN
E2
Hepatitis C virus
Cell adhesion molecules
Endocytosis
HCV genome RNA
E1
E2
NS2
NS3
NS4A
NS4B
NS5A
NS5B
Replication complex
Endoplasmic reticulum
RNA replication
Virion packaging and assembly
Virion maturation and release
Translation and polyprotein processing
Control of replication
2'-5'OAS
2'-5'oligo(A)
RNaseL
mRNA degradation
Inhibition of protein synthesis
Inhibition of virus replication
RIG-I-like receptor signaling pathway
RIG-I
IPS-1
TRAF3
TBK1
IKKε
NS3/4A
IRF3/7
DNA
IFN-α/β
IFN-γ
TNFα
CXCL10
Endosome
TLR3
TRIF
TRAF6
RIP1
IKK
NFκB
IκB
Toll-like receptor signaling pathway
Evasion of innate antiviral immunity
PKR
ISGp56
eIF3
eIF2α
Control of translation
Core
STAT3
SOCS3
IFNα/β
IFNAR1
IFNAR2
Jak1/Tyk2
STAT1/2
IRF9
Jak-STAT signaling pathway
PP2A
PIAS1
Interferon stimulated genes
2'-5'OAS
IRF-7
MxA
Viperin
14-3-3
MAPK signaling pathway
EGF
EGFR
GRB2
SOS
Ras
Raf
MEK
ERK1/2
Cell proliferation
PI3K
PIP3
Akt
GSK3β
β-catenin
Bad
p21
Anti-apoptosis
p53
Cell cycle
CDKs
Rb
E2F
Cell growth
PPAR signaling pathway
RXRα
PPARα
PA28γ
LXRα
CyclinD1
CDK4
c-Myc
Hepatocellular carcinoma, Steatosis
TNF signaling pathway
TNFα
TNFR
TRADD
TRAF2
FasL
Fas
FADD
Casp8
Casp3
c-FLIP
tBid
Bax
Bak
CytC
Casp9
Apaf1
Apoptosis
05160 12/19/18
(c) Kanehisa Laboratories

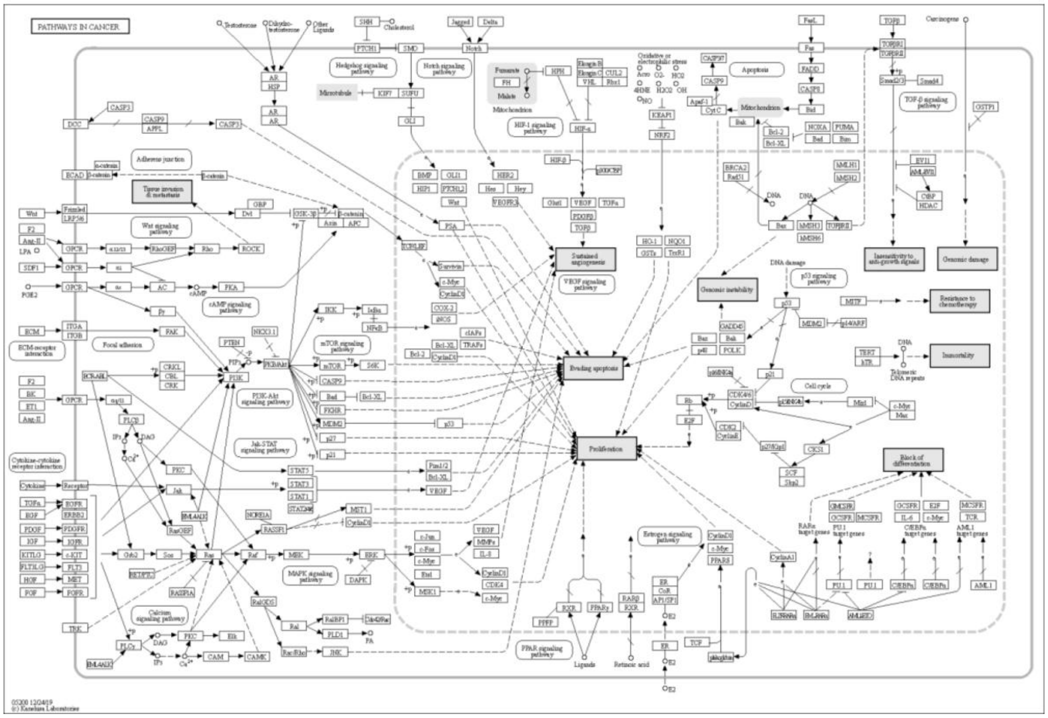
PATHWAYS IN CANCER
05200 12/24/19
(c) Kanehisa Laboratories

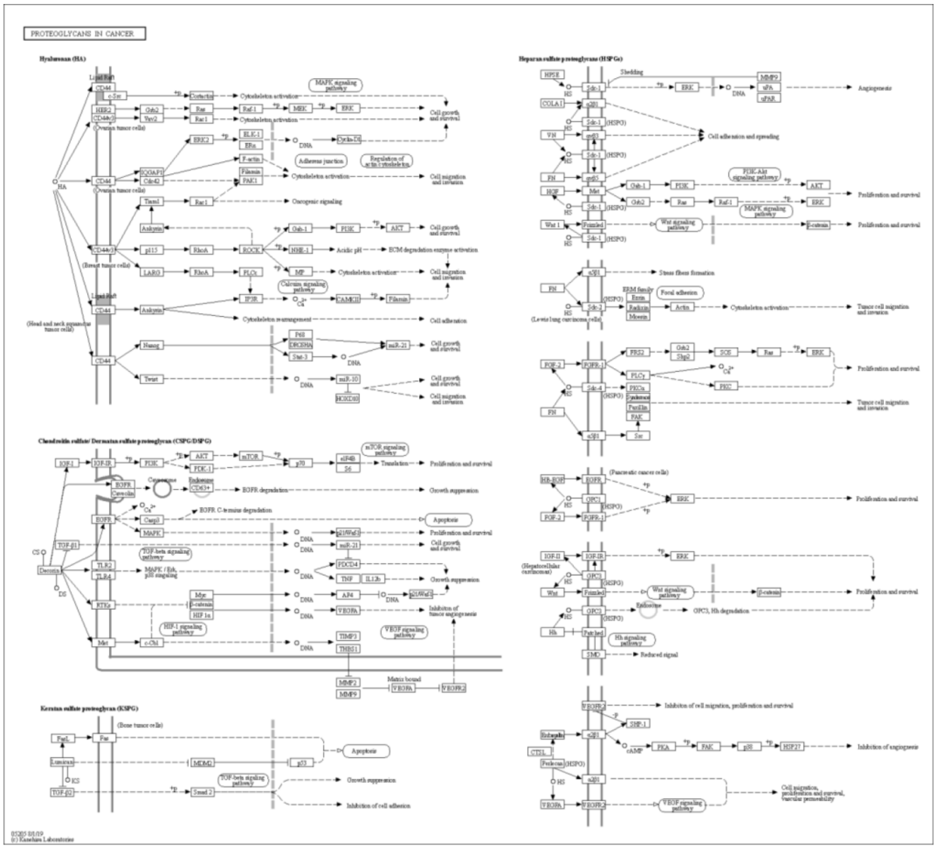
PROTEOGLYCANS IN CANCER

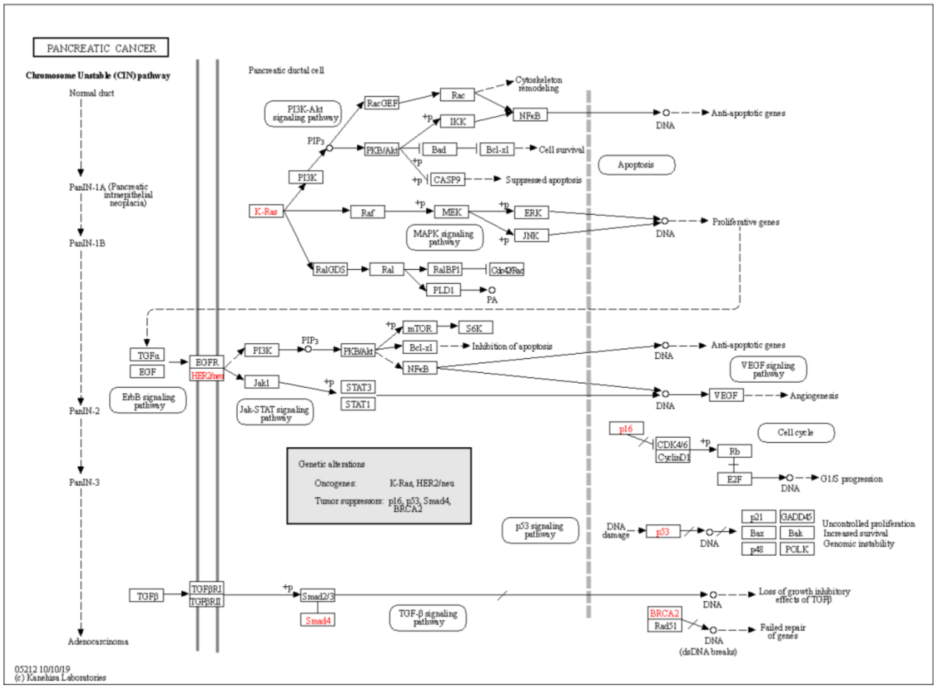
PANCREATIC CANCER
Chromosome Unstable (CIN) pathway
Normal duct
PanIN-1A (Pancreatic intraepithelial neoplasia)
PanIN-1B
PanIN-2
PanIN-3
Adenocarcinoma
Pancreatic ductal cell
PI3K-Akt signaling pathway
RacGEF
Rac
Cytoskeleton remodeling
NFκB
IKK
PIP3
PKB/Akt
Bad
Bcl-xl
Cell survival
CASP9
Suppressed apoptosis
Apoptosis
Anti-apoptotic genes
DNA
PI3K
K-Ras
Raf
MEK
ERK
JNK
MAPK signaling pathway
Proliferative genes
RalGDS
Ral
RalBP1
Cdc42/Rac
PLD1
PA
mTOR
S6K
Bcl-xl
Inhibition of apoptosis
TGFα
EGF
EGFR
HER2/neu
Jak1
STAT3
STAT1
VEGF signaling pathway
VEGF
Angiogenesis
ErbB signaling pathway
Jak-STAT signaling pathway
Genetic alterations
Oncogenes: K-Ras, HER2/neu
Tumor suppressors: p16, p53, Smad4, BRCA2
p16
CDK4/6
CyclinD1
Rb
E2F
Cell cycle
G1/S progression
p53 signaling pathway
DNA damage
p53
p21
GADD45
Bax
Bak
p48
POLK
Uncontrolled proliferation
Increased survival
Genomic instability
TGFβ
TGFβRI
TGFβRII
Smad2/3
Smad4
TGF-β signaling pathway
Loss of growth inhibitory effects of TGFβ
BRCA2
Rad51
Failed repair of genes
(dsDNA breaks)
05212 10/10/19
(c) Kanehisa Laboratories

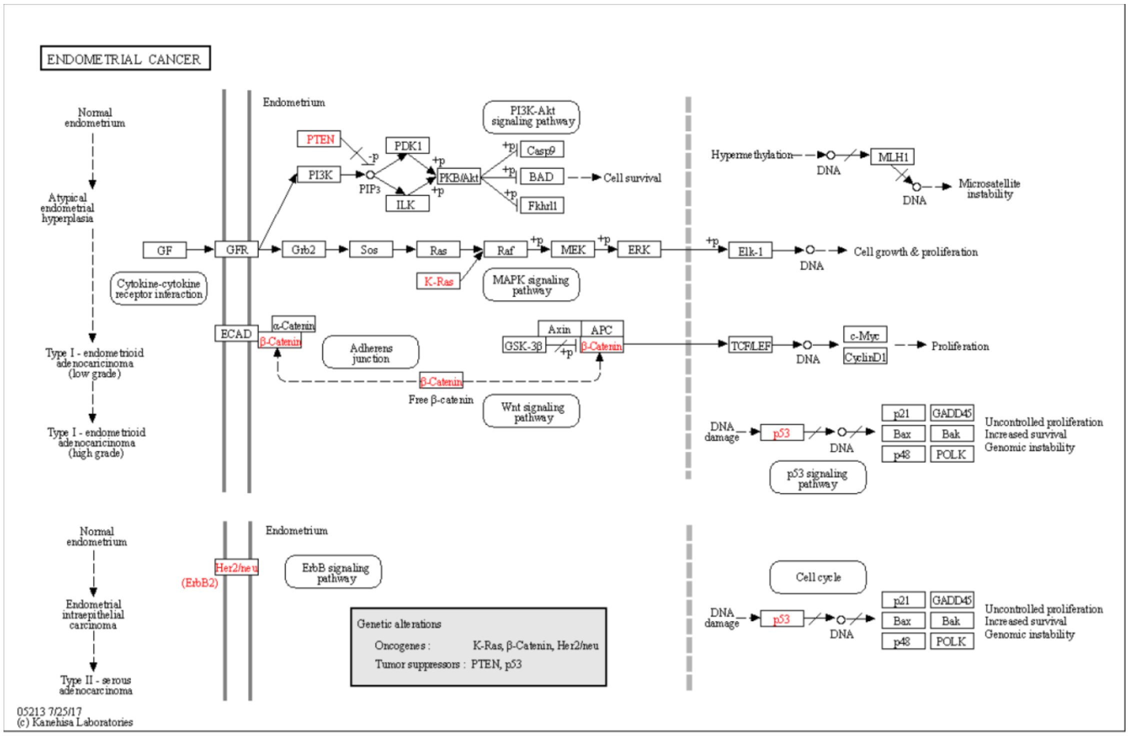
ENDOMETRIAL CANCER
Normal endometrium
Atypical endometrial hyperplasia
Type I - endometrioid adenocarcinoma (low grade)
Type I - endometrioid adenocarcinoma (high grade)
Endometrium
PI3K-Akt signaling pathway
PTEN
PDK1
PI3K
PIP3
ILK
PKB/Akt
Casp9
BAD
Fkhrl1
Cell survival
GF
GFR
Grb2
Sos
Ras
Raf
MEK
ERK
K-Ras
MAPK signaling pathway
Cytokine-cytokine receptor interaction
ECAD
α-Catenin
β-Catenin
Adherens junction
Axin
APC
GSK-3β
Free β-catenin
Wnt signaling pathway
Hypermethylation
DNA
MLH1
Microsatellite instability
Elk-1
Cell growth & proliferation
TCF/LEF
c-Myc
CyclinD1
Proliferation
DNA damage
p53
p21
GADD45
Bax
Bak
p48
POLK
Uncontrolled proliferation
Increased survival
Genomic instability
p53 signaling pathway
Normal endometrium
Endometrial intraepithelial carcinoma
Type II - serous adenocarcinoma
Her2/neu
(ErbB2)
ErbB signaling pathway
Cell cycle
Genetic alterations
Oncogenes : K-Ras, β-Catenin, Her2/neu
Tumor suppressors : PTEN, p53
05213 7/25/17
(c) Kanehisa Laboratories

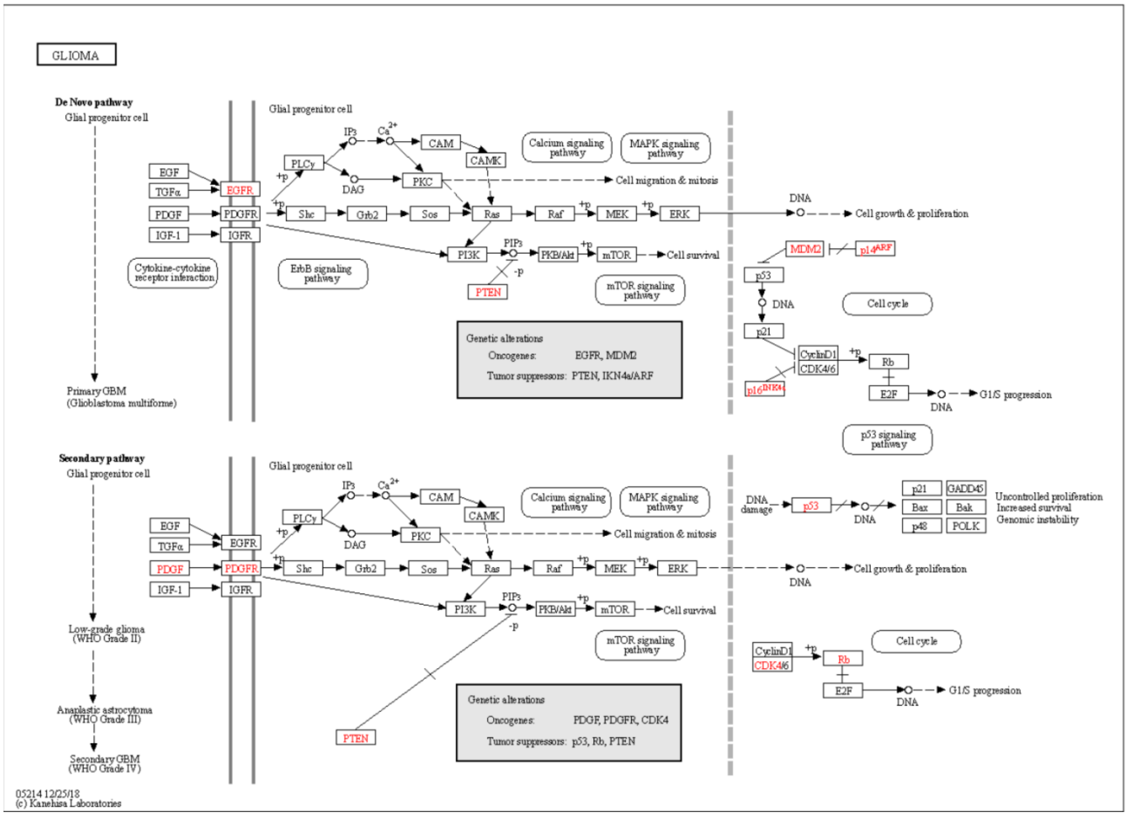
GLIOMA
De Novo pathway
Glial progenitor cell
Primary GBM (Glioblastoma multiforme)
EGF
TGFα
EGFR
PDGF
PDGFR
IGF-1
IGFR
Cytokine-cytokine receptor interaction
ErbB signaling pathway
PLCγ
IP3
Ca2+
CAM
CAMK
DAG
PKC
Calcium signaling pathway
MAPK signaling pathway
Cell migration & mitosis
Shc
Grb2
Sos
Ras
Raf
MEK
ERK
DNA
Cell growth & proliferation
PI3K
PIP3
PKB/Akt
mTOR
Cell survival
PTEN
mTOR signaling pathway
MDM2
p14ARF
p53
p21
Cell cycle
CyclinD1
CDK4/6
Rb
E2F
p16INK4
G1/S progression
p53 signaling pathway
Genetic alterations
Oncogenes: EGFR, MDM2
Tumor suppressors: PTEN, IKN4a/ARF
Secondary pathway
Glial progenitor cell
Low-grade glioma (WHO Grade II)
Anaplastic astrocytoma (WHO Grade III)
Secondary GBM (WHO Grade IV)
DNA damage
GADD45
Bax
Bak
p48
POLK
Uncontrolled proliferation
Increased survival
Genomic instability
Genetic alterations
Oncogenes: PDGF, PDGFR, CDK4
Tumor suppressors: p53, Rb, PTEN
05214 12/25/18
(c) Kanehisa Laboratories

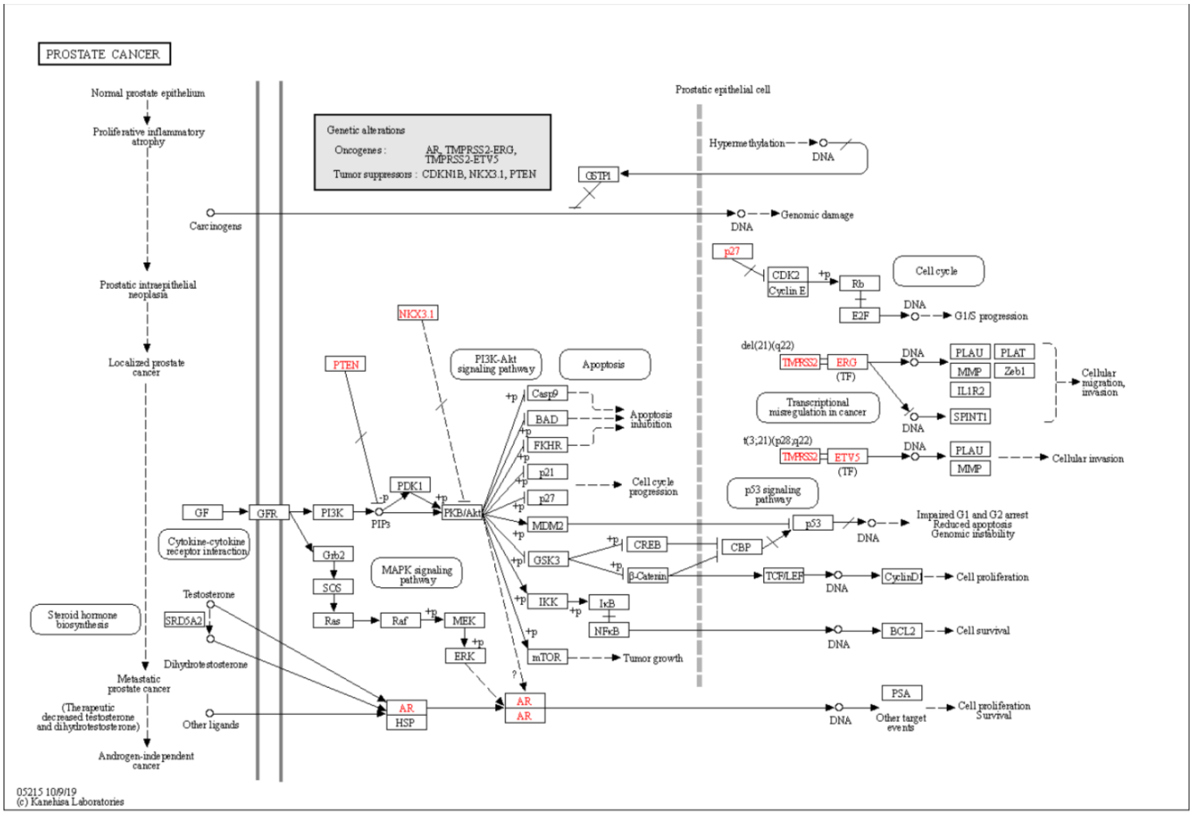
PROSTATE CANCER
Normal prostate epithelium
Proliferative inflammatory atrophy
Prostatic intraepithelial neoplasia
Localized prostate cancer
Metastatic prostate cancer
(Therapeutic decreased testosterone and dihydrotestosterone)
Androgen-independent cancer
Prostatic epithelial cell
Genetic alterations
Oncogenes : AR, TMPRSS2-ERG, TMPRSS2-ETV5
Tumor suppressors : CDKN1B, NKX3.1, PTEN
Hypermethylation
DNA
GSTP1
Carcinogens
Genomic damage
p27
CDK2
Cyclin E
Rb
E2F
Cell cycle
G1/S progression
NKX3.1
PTEN
PI3K-Akt signaling pathway
Apoptosis
del(21)(q22)
TMPRSS2
ERG
(TF)
PLAU
PLAT
MMP
Zeb1
IL1R2
SPINT1
Cellular migration, invasion
Transcriptional misregulation in cancer
Casp9
BAD
FKHR
p21
p27
Apoptosis inhibition
Cell cycle progression
t(3;21)(p28;q22)
ETV5
Cellular invasion
p53 signaling pathway
GF
GFR
PI3K
PIP3
PDK1
PKB/Akt
MDM2
p53
Impaired G1 and G2 arrest
Reduced apoptosis
Genomic instability
Cytokine-cytokine receptor interaction
Grb2
SOS
Ras
Raf
MEK
ERK
MAPK signaling pathway
GSK3
CREB
CBP
β-Catenin
TCF/LEF
CyclinD1
Cell proliferation
IKK
IκB
NFκB
BCL2
Cell survival
Steroid hormone biosynthesis
SRD5A2
Testosterone
Dihydrotestosterone
mTOR
Tumor growth
AR
HSP
Other ligands
PSA
Other target events
Cell proliferation
Survival
05215 10/9/19
(c) Kanehisa Laboratories

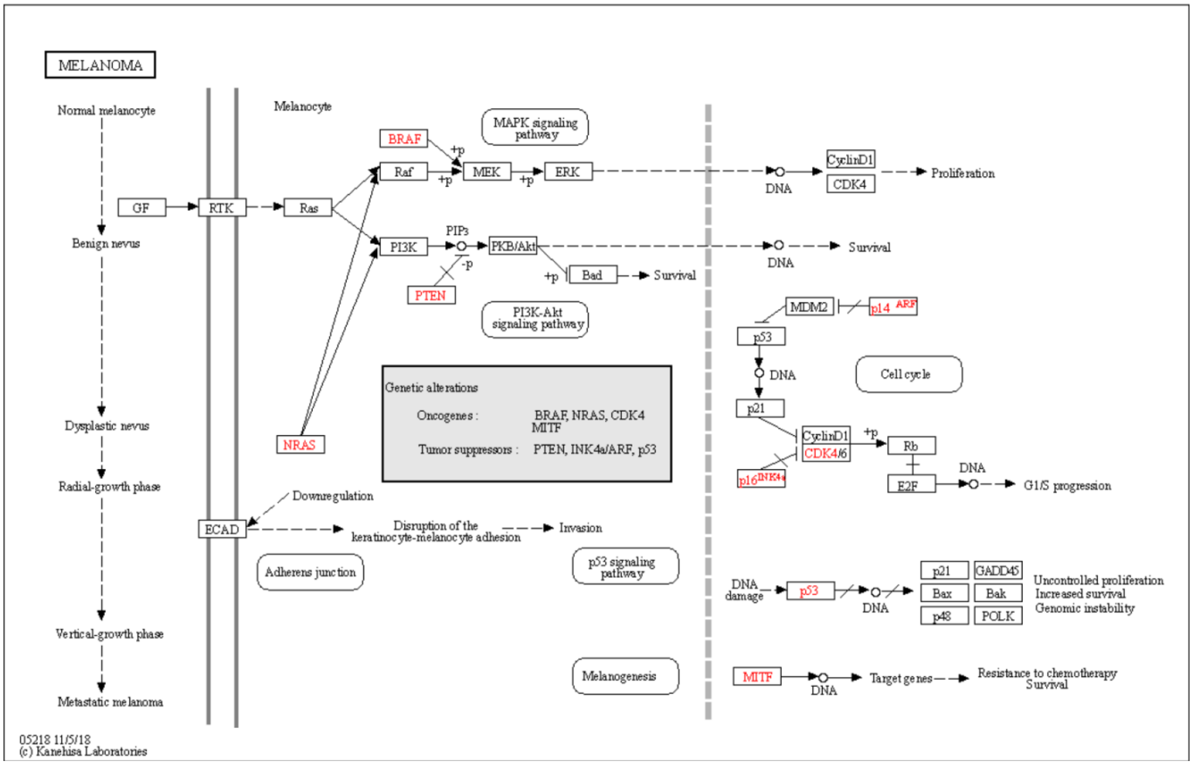
MELANOMA
Normal melanocyte
Benign nevus
Dysplastic nevus
Radial-growth phase
Vertical-growth phase
Metastatic melanoma
Melanocyte
GF
RTK
Ras
BRAF
Raf
MEK
ERK
MAPK signaling pathway
DNA
CyclinD1
CDK4
Proliferation
PI3K
PIP3
PKB/Akt
PTEN
Bad
Survival
PI3K-Akt signaling pathway
MDM2
p14 ARF
p53
p21
CyclinD1
CDK4/6
p16INK4a
Rb
E2F
Cell cycle
G1/S progression
Genetic alterations
Oncogenes : BRAF, NRAS, CDK4 MITF
Tumor suppressors : PTEN, INK4a/ARF, p53
NRAS
ECAD
Downregulation
Disruption of the keratinocyte-melanocyte adhesion
Invasion
Adherens junction
p53 signaling pathway
DNA damage
GADD45
Bax
Bak
p48
POLK
Uncontrolled proliferation
Increased survival
Genomic instability
Melanogenesis
MITF
Target genes
Resistance to chemotherapy
Survival
05218 11/5/18
(c) Kanehisa Laboratories

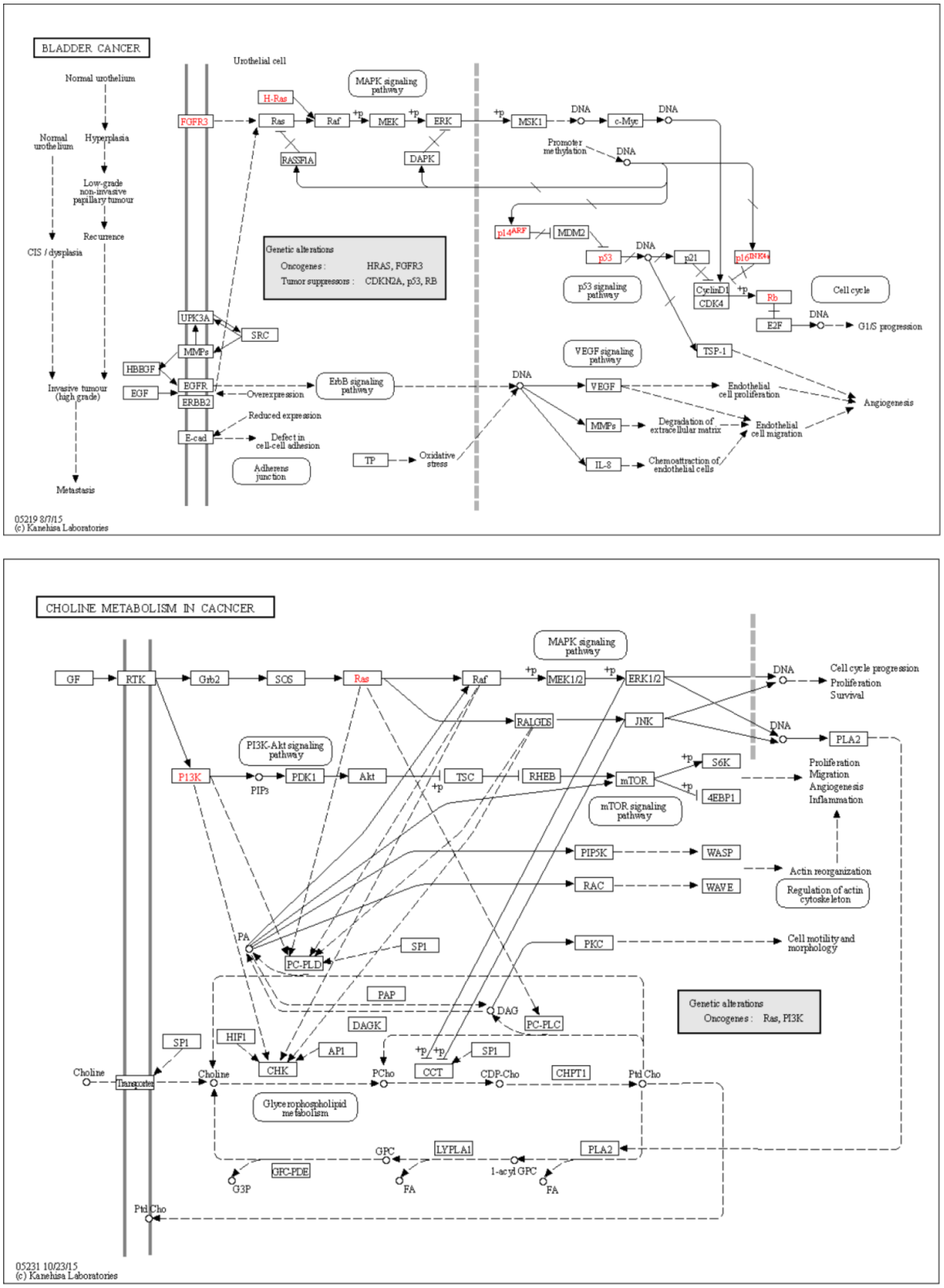

Supplementary Figure 1. More other molecular pathways of the core targets.
